# Android malware analysis in a nutshell

**DOI:** 10.1371/journal.pone.0270647

**Published:** 2022-07-05

**Authors:** Iman Almomani, Mohanned Ahmed, Walid El-Shafai

**Affiliations:** 1 Security Engineering Lab, Computer Science Department, Prince Sultan University, Riyadh, KSA; 2 Computer Science Department, King Abdullah II School of Information Technology, The University of Jordan, Amman, Jordan; 3 Electronics and Electrical Communication Engineering Department, Faculty of Electronic Engineering, Menoufia University, Menouf, Egypt; Hanyang University, KOREA, REPUBLIC OF

## Abstract

This paper offers a comprehensive analysis model for android malware. The model presents the essential factors affecting the analysis results of android malware that are vision-based. Current android malware analysis and solutions might consider one or some of these factors while building their malware predictive systems. However, this paper comprehensively highlights these factors and their impacts through a deep empirical study. The study comprises 22 CNN (Convolutional Neural Network) algorithms, 21 of them are well-known, and one proposed algorithm. Additionally, several types of files are considered before converting them to images, and two benchmark android malware datasets are utilized. Finally, comprehensive evaluation metrics are measured to assess the produced predictive models from the security and complexity perspectives. Consequently, guiding researchers and developers to plan and build efficient malware analysis systems that meet their requirements and resources. The results reveal that some factors might significantly impact the performance of the malware analysis solution. For example, from a security perspective, the accuracy, F1-score, precision, and recall are improved by 131.29%, 236.44%, 192%, and 131.29%, respectively, when changing one factor and fixing all other factors under study. Similar results are observed in the case of complexity assessment, including testing time, CPU usage, storage size, and pre-processing speed, proving the importance of the proposed android malware analysis model.

## Introduction

**Mal**cious soft**ware** (Malware) is any software built for unauthorized purposes and mala fide aims. So, the malware affects the operating system performance and its running services due to its harmful behavior. Currently, android malware is one of the most critical threats that can encrypt or defect the operation of Android devices [1]. This is because Android malware applications (APKs) can steal or cipher sensitive data, show undesirable advertising, disrupt normal functions, or control the users’ devices without their knowledge [2].

There are a lot of groups and categories of Android malware APKs, such as worms, botnet, rootkits, ransomware, and Trojans [3]. These Android malware attacks can exploit metamorphic and polymorphic procedures to obfuscate traditional malware recognition and detection algorithms. Moreover, the Android malware developers have a tendency to modify small sections of the developed and implemented source codes to create other malware alternatives and threats that can evade the malware detection techniques [4]. Consequently, the identification process of Android malware attacks from the same malware family becomes tremendously challenging [5]. Therefore, efficient Android malware detection algorithms based on smart artificial intelligence (AI) tools need to be developed and implemented to identify and recognize the harmful effect of Android malware threats [6, 7].

Android malware detection and identification algorithms are categorized into four main groups: static-based, dynamic-based, vision-based, or hybrid-based detection algorithms [8–12]. In static-based identification algorithms, the Android malware APKs are analyzed without executing them. So, these static-based algorithms depend on extracting some of the important features from the suspected source codes to identify and recognize the Android malware families. However, the main disadvantage of these static-based algorithms is that they are not robust to code obfuscation, and they need more computation steps during the process of extracting features [13, 14]. In dynamic-based identification algorithms, the traces and features of the suspected source codes are examined and analyzed during their execution and running. The critical disadvantage of these algorithms is that they are more time-consuming and require additional storage resources [15].

On the other hand, in the hybrid-based identification algorithms, two or more types of identification categories are simultaneously employed to efficiently detect the Android malware attacks. But this malware identification category needs more sequential steps, high computational complexity, human intervention, and manual effort [16]. In vision-based malware identification algorithms, the Android malware APKs or their extracted features are converted to visual 2D digital images before the classification and detection process. Therefore, the main features of the Android malware APKs can be extracted and obtained by the unzipping or decompilation processes [17, 18]. Then, the resulting 1D binary vectors of the extracted features (i.e., Android manifest, SMALI, and Classes.dex) are transformed to 2D vectors (grayscale images). In the last step, the resulting 2D grayscale images are forwarded to a well-developed malware classifier such as Convolutional Neural Networks (CNN)-based malware classifiers to detect and classify the category and family of the analyzed Android malware APKs.

Recently, Deep Learning (DL) and optimization algorithms are currently utilized and exploited in mitigating Android malware threats [19–22]. Thus, DL networks such as CNN algorithms are the most common AI and DL-based recognition & identification techniques used to detect malware attacks from the input malware visual images [23–25]. Furthermore, the CNN networks have the ability to efficiently distinguish various objects and aspects on the input visual images using well-tuned learning biases and weights based on utilizing optimization algorithms. Therefore, the CNN algorithms are the best choice for image classification challenges and applications, such as classifying malware images [26–29]. Consequently, efficient developed CNN algorithms can be used to automatically collect and obtain the rich and valuable features from Android malware visual images. Then, these obtained features are used to classify and identify the different families of Android malicious APKs.

Therefore, in our proposed work, without executing or running the Android APKs, we first converted their binary data into 2D images. After that, we employed a well-developed CNN-based Android malware detection algorithm to classify different categories of Android malware families from these 2D images. In addition, we tested and analyzed different 21 pre-trained CNN algorithms to check their detection performance in identifying and recognizing the Android malware classes from their visual images. Thus, the DL-based CNN algorithms differ from traditional Machine Learning (ML) algorithms that accomplish feature representation with specific parameter configuration or particular assumptions. Therefore, compared to conventional ML algorithms, the DL-based CNN algorithms can effectively discover complex patterns and obtain valuable features from multi-dimensional patterns like visual images.

In Android malware analysis and detection systems, many parameters and factors need to be considered that control the identification and recognition performance of the utilized malware classifiers. These parameters include (1) the analyzed Android dataset (balanced or imbalanced), (2) the utilized evaluation metrics (i.e., security or complexity metrics), (3) the type of malware analysis (static, dynamic, hybrid, or vision), and (4) the type of APK components selected to be analyzed in the detection process (i.e., Full APK file, android manifest file, SMALI file, or Classes.dex file).

So, this research is motivated by the importance of the area of android malware analysis and detection solutions due to the increased risk of such types of attacks. In addition, there are tremendous existing efforts utilizing vision-based algorithms to analyze and detect android malware with high accuracy. The most critical issue in the previous related works is that they only studied some parameters in their introduced malware detection systems. But, to achieve high detection accuracy and efficient malware analysis, many factors must be investigated that directly or indirectly affect the malware classification process.

As most current malware detection systems consider one or some factors while building their malware predictive systems, this motivates us to offer a comprehensive analysis model for Android malware. The model presents the essential factors affecting the analysis results of vision-based Android malware. Consequently, we comprehensively highlighted these factors and tested their impacts through a deep empirical study. The goal is to support researchers and developers by providing a clear guide on planning and building efficient Malware analysis systems that meet their requirements and available resources.

The significant contributions of our work are detailed as follows:

- Summarizing and comparing the most recent vision-based Android malware detection systems and the main factors studied by them.
- Proposing a nutshell vision-based model for efficiently detecting malware apps. This model considers a comprehensive set of factors that might impact the efficiency of malware analysis and detection solutions from the security and complexity perspectives. These factors include the nature of the malware datasets, APK conversion process & format, CNN algorithms used, and the evaluation metrics applied.
- Constructing a deep empirical study to implement all these factors and related parameters and analyze their impacts by running more than 450 experiments within the same environmen.
- Investigating the malware detection performance of different 22 CNN algorithms as part of the empirical study on the two most common imbalanced Android malware datasets (DREBIN and AMD). One of these CNN algorithms is developed from scratch for this research.
- Avoiding the need for static or dynamic analysis for classifying Android malware attacks by converting Android threats to visual images for easy and low-complex classification process using CNN algorithms. Thus, we achieved low computational complexity and, at the same time, obtained high detection accuracy.
- Studying the impact of different visual formats of Android malware APKs on the security and complexity performance of malware detection algorithms.
- Analyzing highly imbalanced Android malware datasets containing unbalanced malware classes to achieve proper detection performance.
- Report and analyze the experiments’ results whether the malware APKs were directly converted to images or rich extracted features from the Android APKs were converted to visual images.
- Performing a deep comparative analysis for the security and complexity metrics performance of all tested scenarios composed in the proposed comprehensive vision-based model.

The structure of this work is as follows. Section Related Work summarizes and compares the recent related studies. Section Proposed presents the proposed comprehensive android malware analysis model. Section Analysis illustrates the model evaluation and results discussions & analysis. Finally, Section Conclusions concludes the paper and offers some future directions.

## Related work

This section summarizes and compares the previous work related to image-based malware detection algorithms and systems. Table 1 shows a summary and comparison in terms of type of image conversion (and if the process used involves unzipping, de-compilation, or both), used dataset(s), utilized CNN algorithms, performance evaluation measures considered such as model/prepossessing complexity and security measures.

**Table 1:** Summary of recent related vision-based Android malware detection systems.

Ref	Type of Image Conversion	Dataset(s)	CNN Algorithms	Performance Evaluation	
				Model/Preprocessing Complexity Measures	Security Measures
[30]	unzipping: DEX file to gray markov image	DREBIN	CNN-VGG16	None	Accuracy, Precision Recall, F1-score
[31]	unzipping: DEX \ resource \ manifest \ certificate files to gray image	DREBIN	CNN-SVM CNN-KNN CNN-RF VGG16	RAM Usage Training Time Preprocessing Time	Accuracy Precision, Recall Error Rate, MSE
[35]	de-compilation: raw opcodes to image	DREBIN AMD	CNN	Test Time Training Time Preprocessing Time	Accuracy, Precision Recall, F1-score
[32]	unzipping: DEX \ AM files to gray /color image	DREBIN AMD	CNN-ResNet	Preprocessing Time Training Time	TP,TN,FN,FP Accuracy
[36]	de-compilation: Opcodes \ API packages \ high level risky API functions to RGB image	DREBIN	CNN	None	Accuracy TPR,FPR Precision,F1-score
[37]	de-compilation: Permisions to image	DREBIN AMD	CNN	Training Time	Accuracy Sensitivity, Specificity Precision, F1-score
[33]	unzipping: AM \ DEX\ Resource.ARSC to gray image	DREBIN AMD	RF KNN DT Bagging AdaBoost Gradient Boost	Test Time Training Time Preprocessing Time	Accuracy
[39]	network interactions to gray image	AMD	CNN	APK File Size	TP,TN,FN,FP Accuracy, Precision Recall, F1-score
[34]	unzipping: AM \ DEX \ Certificates to RGB images	DREBIN	VGG16	None	Accuracy, Precision Recall, F1-score
**Ours**	**de-compilation & unzipping**: **APK,AM** **DEX, SMALI** **DAM to gray image**	**DREBIN** **AMD**	**22 CNN Models**	**Training Time** **Testing Time** **RAM Usage** **Preprocessing Time** **File size of APK image DEX image, AM image DAM image, SMALI image**	**TP,TNR,FN,FP** **Accuracy** **Precision** **Recall** **F1-score** **TN,NPV,FPR,FNR** **PPV,FDR,FOR,MR**

Various algorithms were introduced in the literature that use unzipping, de-compilation, or both in the image conversion process. Regarding unzipping-related approaches [30], introduced a byte-level malware classification method by using Markov technique in classes.dex-to-image conversion and then using deep CNN for the classification. Moreover [31], proposed a system to classify malware by converting non-intuitive features into images to extract features using CNN and use the features in classical ML algorithms such as KNN to detect the malware family. [32] implemented and introduced a color visualization method on classes.dex and AndroidManifest.xml files in malware Android apps and classify the images using CNN-ResNet models. In [33] paper, classical machine algorithms such as Random Forest, K-nearest Neighbors, Decision Tree, Bagging, AdaBoost, and Gradient Boost were used for classification after constructing feature vectors from gray images, yielded from converting APK contents such as classes.dex to images. [34] proposed an approach to enhance blockchain user security by implementing RGB image visualization technique on three types of files in Android apps: classes.dex, AndroidManifest.xml, and Certificate. Then, train different classification models and apply a decision mechanism to detect malware versus benign. On the other hand, for de-compilation techniques [35], introduced a method called AdMat which treats Android apps as images by forming an adjacency matrix for each app and then feeding them to the CNN model to classify an app to malware or benign. Additionally [36], combined Opcodes, API packages, and API functions to construct RGB images and then use CNN for classification. [37] mapped permissions to severity levels [38]to create images to be fed to the CNN model for malware classification. Other methods such as [39] used network interactions as features to be converted to images to be input for CNN.

Different datasets were used in the previous papers to test the models and systems. The main ones were Drebin and AMD. Some of them used DREBIN alone, such as [31, 36], and some of them used only AMD, such as [39]. However, most of them used a combination of both [32, 33, 35, 37].

To evaluate the performance of the resulted predictive models, several metrics were used in the literature. Common metrics were accuracy, precision, recall, and F1-score [30, 31, 34–36]. Other metrics were used such as error rate, specificity, sensitivity, MSE, and FPR [31, 32, 36, 37].

Even though different works have been introduced for malware detection analysis, none of them studied the approach comprehensively in terms of the used image conversion methods, datasets, CNN models, and evaluation metrics. This can be clearly observed in the comparison conducted among the related work and our proposed analysis model, as shown in Table 1. For example, in terms of the employed CNN algorithms used, most of the related works examined a few models such as VGG16, ResNet, and customized CNN algorithms such as in [30–32, 34–37, 39]. Moreover, they did not take into consideration all different file formats of Android malware samples. For instance, the authors in [30–34] focused on the unzipping prepossessing without considering the impact of decompiling preprocessing for the Android malware APKs. On the other hand, the authors in [35–37] only considered the decompiling preprocessing. Additionally, few assessment metrics were used for performance evaluation and complexity & security analysis of the examined CNN algorithms. For example, some of the related studies used training time, prepossessing time, test time, APK file size, and RAM usage as complexity parameters, such as in [30–32, 34–37, 39]. However, none of these related works introduced a comprehensive analysis of all of these complexity metrics. Moreover, in terms of security measures used, the related works used various security metrics such as accuracy, precision, recall, and F1-score, such as in [30–32, 34–37, 39]. But many other assessment metrics must be considered and analyzed. For instance, the authors in [31] considered other metrics such as error rate and MSE, while the authors in [36] evaluated their suggested CNN algorithms using TPR and FPR. However, most related studies did not present deep and comprehensive security analyses such as the estimation of NPV, PPV, and FOR parameters that can provide more insights.

Therefore, in this paper, we introduce a comprehensive model that profoundly investigates the critical factors that might impact the performance of android malware analysis systems in terms of efficiency, complexity, and security perspectives. Our proposed work covers different APK file formats, and different scenarios of de-compilation & unzipping preprocessing to extract more features from the android APKs such as AM, DEX, de-compiled AM, and SMALI. Additionally, the proposed Android malware analysis model tests the performance of different 22 CNN algorithms in terms of comprehensive security and complexity metrics to deeply analyze their detection and computational efficiencies.

## Proposed comprehensive Android malware analysis model

**Fig 1:**
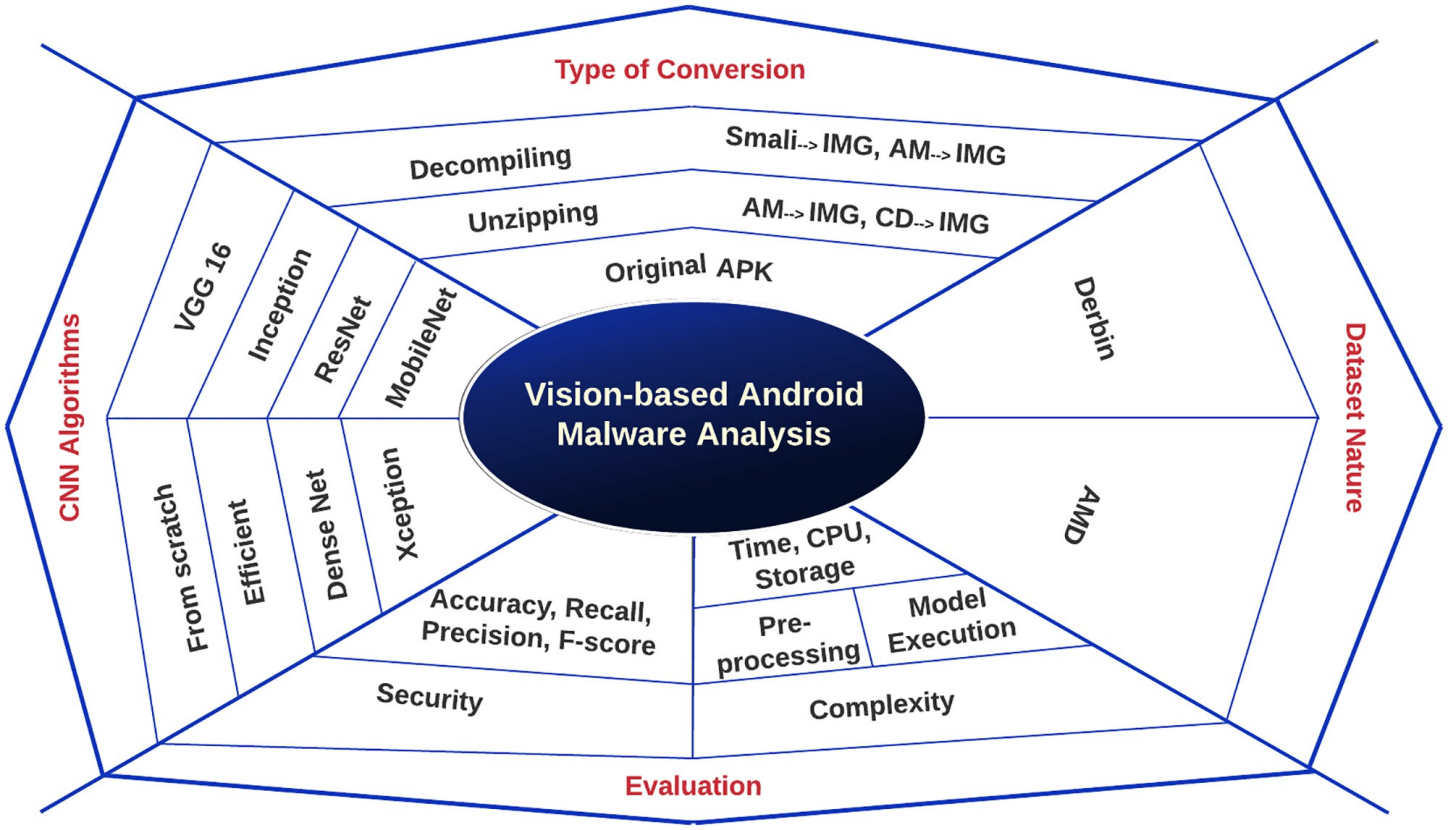
The high-level description of the in-detail processes in the proposed comprehensive model.

This section presents a nutshell model of building vision-based prediction models for Android malware detection systems. As shown in Fig 1, primary factors should be considered as they will affect the Android malware analysis and detection processes. These factors include:

- **Type of conversion**: This defines how the Android malware apk is analyzed. One option is to keep it as is (compressed) and then convert it to an image. Another option is to decompile the apk file first using different tools such as apktool (https://ibotpeaches.github.io/Apktool/). This tool decompiles the apk to generate smali files and the Android Manifest (AM) file. Then these files will be stacked and then converted to images. Additionally, the analysis system could consider only unzipping the apk file and then converting the resulting AM file and “classes.dex” (CD) files to images.
- **Dataset Nature**: The created or chosen Android malware dataset could severely impact the analysis model and the resulted predictive models. This includes the type of malware apps considered and their primary behavior, the number of families (classes), and whether the dataset is balanced or not.
- **CNN Algorithms**: The type of CNN algorithm that will be used to build the predictive model is vital to the performance of the malware detection systems. Therefore, this study has examined most of the well-known CNN algorithms (all currently implemented by Keras (https://keras.io/)) to provide a deep insight into the CNN algorithms’ impact on detecting Android malware applications.
- **Evaluation**: The way the Android malware analysis and predictive models are evaluated is critical to trade-off the system performance in terms of security and complexity. Therefore, the evaluation metrics must be carefully selected based on the system’s needs and available resources.

**Fig 2:**
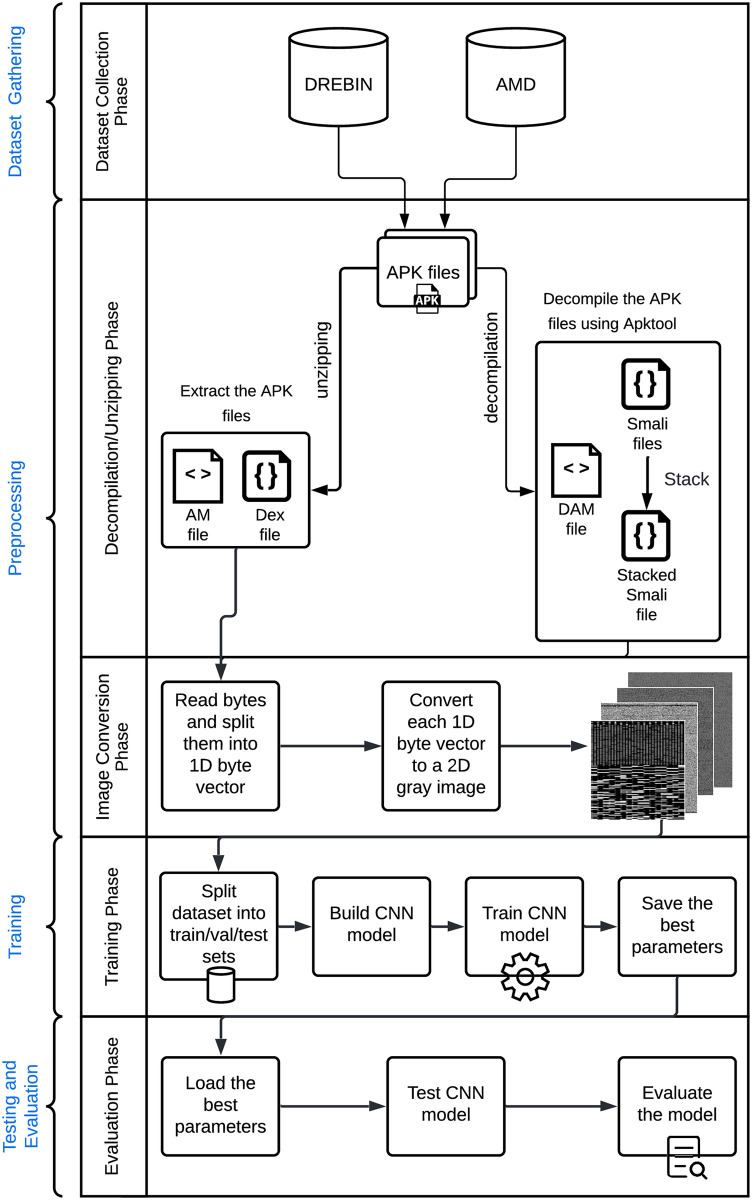
The flow of the proposed comprehensive model.

The main flow of our proposed model is illustrated in Fig 2. The first phase in the proposed nutshell model is the selection of the benchmarked android applications (apks) datasets that are heavily utilized in vision-based malware analysis systems. Therefore, both DREBIN [40] and AMD [41] have been selected. The reason behind choosing two different datasets is to show the impact of only changing the nature of the dataset that the system is analyzing and testing on the performance of the overall detection process. After that, the model processes these apks in different ways of conversions: (1) apk is kept compressed as is, (2) apk is decompiled using Apktool to produce android decompiled manifest file (DAM), and Smali files, and (3) apk is unzipped to generate android manifest (AM) file and Dex files. Then, the image conversion phase is started by converting all features resulting from the above files into images. These visual malware images are obtained by converting the extracted features’ binaries to 8-bit vectors and then converted to 2D grayscale images. For more details and explanations for the byte-to-image conversion process, it can be checked in [23, 26].

The final phase is applying 22 CNN models for training and testing the predictive models and then evaluating their performances using a comprehensive set of assessment metrics related to complexity such as time, CPU & storage utilization for both the per-processing and model execution phases. Additionally, 16 security-related metrics are also measured.

**Fig 3:**
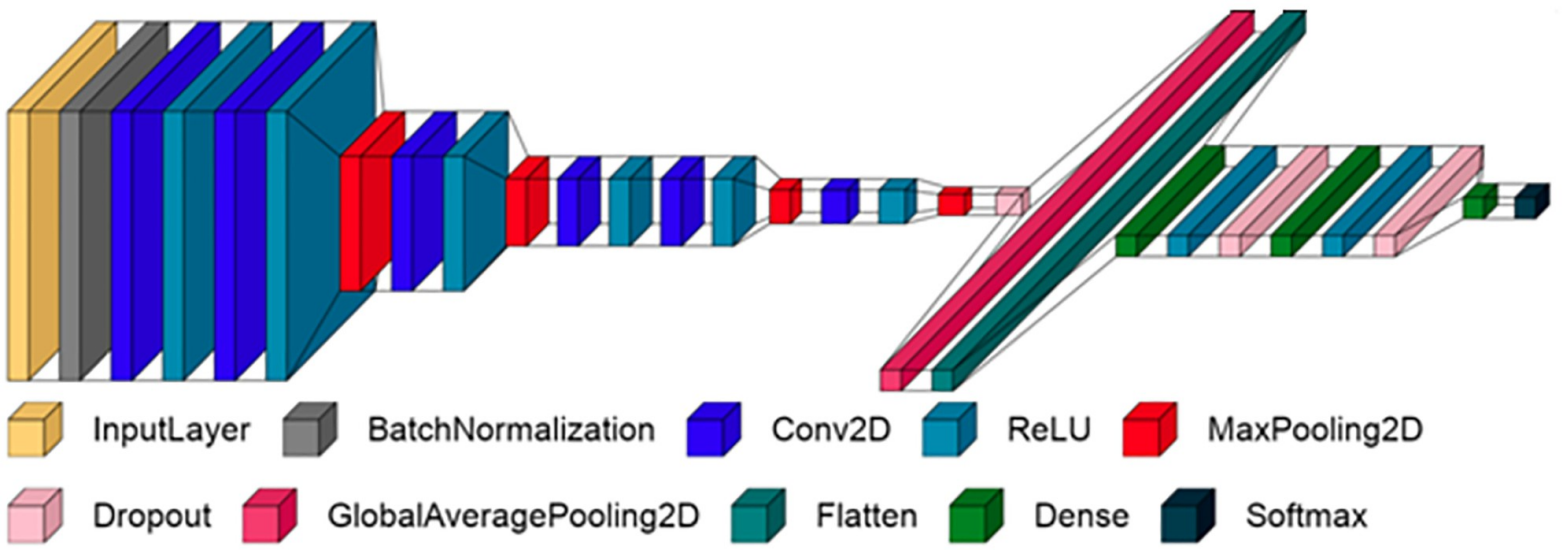
Proposed scratch CNN algorithm.

21 pre-trained CNN algorithms (VGG16, ResNet50, VGG19, DenseNet121, DenseNet169, DenseNet201, EfficientNetB0, EfficientNetB1, EfficientNetB2, EfficientNetB3, EfficientNetB4, EfficientNetB5, EfficientNetB6, EfficientNetB7, InceptionResNetV2, InceptionV3, MobileNet, MobileNetV2, MobileNetV3Large, MobileNetV3Small, and Xception) [42–44] are examined. These pretrained CNN algorithms are developed in Python and implemented in Keras and TensorFlow libraries [45–47]. Additionally, another CNN algorithms is developed from scratch in this research. This algorithm has different layers, as shown in Fig 3.

It consists of several sequential stages. The first stage is the processing of the input visual malware images through the input layer and the Batch-Normalization (BN) layer that normalizes the visual images by re-scaling and re-centering processes. The BN layer is also introduced in the proposed algorithm to stabilize the CNN network. Then, in the second stage, the superlative and furthermost effective features are extracted and accumulated through several 2D convolutional layers (Conv2D), containing the same padding and stride by one. The weights of each utilized Conv2D are initialized with an orthogonal matrix.

The number of employed filters in the Conv2D layers are 8, 16, 32, 64, 64, 256, respectively. Also, the Conv2D layers are interspersed with pooling layers called MaxPooling that selecting the most significant pixel values in a four-pixel space. So, the MaxPooling layers are characterized by reducing the computational burden of the proposed neural CNN network. After that, the GlobalAveragePooling2D is introduced to gather the most common features during the training process.

In the last stage, which is the decision-making, classification & detection stage, the spatial data is primarily converted to one-dimensional data by the flatten later. Next, three sequential fully connected layers (Dense) are utilized, each one of the first two Dense layers consists of 1024 nodes (neurons) whilst the last Dense layer consists of a number of nodes that equal the number of classified classes (eight malware classes in our proposed work). In addition, in the proposed CNN algorithm, we used the Dropout layer to prevent the overfitting problem. Furthermore, the Rectified Linear Unit (ReLU) is also utilized in all Conv2D and Dense layers as an activation function. But the ReLU is used in the last SoftMax layer to make the classification decision. Table 2 presents the specifications of all employed layers in the proposed CNN algorithm.

**Table 2:** Specifications of the CNN layers in the proposed scratch algorithm.

Layer (type)	Output shape	Parameters
batch_normalization (Batch Normalization)	(None, 224, 224, 3)	12
conv2d (Conv2D)	(None, 224, 224, 8)	224
conv2d_1 (Conv2D)	(None, 224, 224, 16)	1168
max_pooling2d (MaxPooling2D)	(None, 112, 112, 16)	0
conv2d_2 (Conv2D)	(None, 112, 112, 32)	4640
max_pooling2d_1 (MaxPooling 2D)	(None, 56, 56, 32)	0
conv2d_3 (Conv2D)	(None, 56, 56, 64)	18496
conv2d_4 (Conv2D)	(None, 56, 56, 64)	36928
max_pooling2d_2 (MaxPooling 2D)	(None, 28, 28, 64)	0
conv2d_5 (Conv2D)	(None, 28, 28, 256)	147712
max_pooling2d_3 (MaxPooling 2D)	(None, 14, 14, 256)	0
dropout (Dropout)	(None, 14, 14, 256)	0
global_average_pooling2d (GlobalAveragePooling2D)	(None, 256)	0
flatten (Flatten)	(None, 256)	0
dense (Dense)	(None, 1024)	263168
dropout_1 (Dropout)	(None, 1024)	0
dense_1 (Dense)	(None, 1024)	1049600
dropout_2 (Dropout)	(None, 1024)	0
dense_2 (Dense)	(None, 10)	10250
**Total params**: 1,532,198, **Trainable params**: 1,532,192, and **Non-trainable params**: 6		

## Model evaluation and results analysis

This section describes and discusses the security and complexity analysis for the proposed comprehensive model. So, the in-detail analysis and testing of the employed 22 CNN algorithms are introduced in terms of different evaluation metrics. The simulation specifications of all examined CNN algorithms in the proposed comprehensive vision-based android malware detection model is summarized in Table 3.

**Table 3:** Simulation specifications of the examined CNN algorithms in the proposed comprehensive model.

Variable	Value
Programming language	Python
Software libraries	Keras and TensorFlow
Training/Validation/Testing ratio	64/16/10 (%)
Rate of the learning process	0.0001
Optimization algorithm	Adam optimizer
Regularization algorithm	L2 regularizer
Decay rate of the regularization algorithm	0.001
Number of epochs	128
Minimum batch size	64
Loss function	Categorical cross-entropy function

Two imbalanced android datasets (DREBIN [40] and AMD [41]) are examined in the simulation analysis. Each one of these datasets contains eight android malware classes. The names and numbers of android malware APKs of the examined DREBIN and AMD datasets are presented in Table 4.

**Table 4:** Description of the examined android malware datasets.

DREBIN		AMD	
Class Name	No. of APKs	Class Name	No. of APKs
FakeInst	925	Dowgin	3385
DroidKungFu	667	FakeInst	2172
Plankton	625	Mecor	1820
Opfake	613	Youmi	1301
GinMaster	339	Fusob	1277
BaseBridge	330	Kuguo	1199
Iconosys	152	BankBot	648
Kmin	147	Jisut	560

### Assessment metrics

Different classification metrics have used to examine the security analysis of the studied CNN algorithms. The mathematical expressions of these evaluation metrics are given as follows:

$$\begin{array}{c} \;\text{Accuracy}\;=\frac{TN+TP}{TN+FP+TP+FN} \end{array}$$ (1)

$$\begin{array}{c} \text{Recall}\;=\frac{TP}{TP+FN} \end{array}$$ (2)

$$\begin{array}{c} \text{Precision}\;\text{(PPV)}\;=\frac{TP}{FP+TP} \end{array}$$ (3)

$$\begin{array}{c} F1\text{-Score}=\frac{2TP}{2TP+FN+FP} \end{array}$$ (4)

$$\begin{array}{c} \text{TNR}\;=\frac{TN}{FP+TN} \end{array}$$ (5)

$$\begin{array}{c} \text{NPV}=\frac{TN}{FN+TN} \end{array}$$ (6)

$$\begin{array}{c} \text{FPR}=\frac{FP}{FP+TN} \end{array}$$ (7)

$$\begin{array}{c} \text{FNR}=\frac{FN}{FN+TP} \end{array}$$ (8)

$$\begin{array}{c} \text{FDR}=\frac{FP}{FP+TP} \end{array}$$ (9)

$$\begin{array}{c} \text{FOR}=\frac{FN}{FN+TN} \end{array}$$ (10)

$$\begin{array}{c} \text{Misclassification}\;\text{rate}\;\text{(MR)}\;=\frac{FP+FN}{TN+FP+TP+FN} \end{array}$$ (11)

where *FP* (false positive), *FN* (false negative), *TP* (true positive), and *TN* (true negative) are calculated by the obtained confusion matrix of the tested CNN algorithm. Where, PPV: positive predictive value, TNR: true negative rate, NPV: negative predictive value, FPR: false positive rate, FNR: false negative rate, TPR: true positive rate, FDR: false discovery rate, and FOR: false omission rate.

### Security analysis

To assess the security of the proposed comprehensive model, we carried out extensive simulation experiments based on different vision-based scenarios. So, the examined 22 CNN algorithms, including the proposed one, are tested on five vision-based formats, which are: (1) direction conversion of an APK file to a visual image, (2) conversion of Android Manifest (AM) file extracted from the unzipping process to a visual image, (3) conversion of AM file extracted from the decompilation (DAM) process to a visual image, (4) conversion of Classes.dex (CD) file extracted from the unzipping process to a visual image, and (5) conversion of SMALI file extracted from the decompilation process to a visual image. All the above-mentioned security-related metrics are calculated. For simplicity in presenting and comparing the results, the accuracy, precision, recall, and F1-Score metrics are highlighted in each tested CNN algorithm for the five studied vision-based scenarios on two different android malware datasets (DREBIN & AMD), as shown in Tables 5 and 6.

**Table 5:** Security performance of models on DREBIN dataset.

Model	Metric	APK	AM	CD	DAM	SMALI
**Ours**	Accuracy(%)	88.77	93.73	94.52	95.82	90.86
	F1-Score(%)	88.75	93.75	94.52	95.78	91.0
	Precision(%)	88.95	93.9	94.64	95.95	91.56
	Recall(%)	88.77	93.73	94.52	95.82	90.86
**VGG16**	Accuracy(%)	84.33	93.99	93.21	92.95	89.03
	F1-Score(%)	84.15	93.86	93.35	92.84	88.64
	Precision(%)	85.05	94.01	93.9	93.13	89.57
	Recall(%)	84.33	93.99	93.21	92.95	89.03
**ResNet50**	Accuracy(%)	86.68	94.78	92.69	95.56	90.6
	F1-Score(%)	86.63	94.74	92.72	95.53	90.66
	Precision(%)	86.92	94.95	92.95	95.71	91.03
	Recall(%)	86.68	94.78	92.69	95.56	90.6
**VGG19**	Accuracy(%)	83.55	93.99	94.52	92.95	89.82
	F1-Score(%)	83.48	94.0	94.61	92.85	89.69
	Precision(%)	83.92	94.28	94.99	93.27	89.8
	Recall(%)	83.55	93.99	94.52	92.95	89.82
**DenseNet121**	Accuracy(%)	79.37	89.82	93.21	93.21	85.64
	F1-Score(%)	79.05	89.98	93.26	93.17	85.59
	Precision(%)	80.41	90.6	93.48	93.64	86.5
	Recall(%)	79.37	89.82	93.21	93.21	85.64
**DenseNet169**	Accuracy(%)	84.07	91.38	91.91	91.38	87.47
	F1-Score(%)	83.86	91.38	92.06	91.28	87.31
	Precision(%)	84.5	91.58	92.76	91.59	87.94
	Recall(%)	84.07	91.38	91.91	91.38	87.47
**DenseNet201**	Accuracy(%)	84.33	88.25	92.69	92.69	88.77
	F1-Score(%)	83.96	87.95	92.81	92.56	88.79
	Precision(%)	84.91	88.68	93.18	92.63	89.04
	Recall(%)	84.33	88.25	92.69	92.69	88.77
**EfficientNetB0**	Accuracy(%)	83.55	93.47	91.91	92.17	88.51
	F1-Score(%)	83.38	93.46	91.95	92.12	88.41
	Precision(%)	83.84	93.69	92.24	92.44	88.62
	Recall(%)	83.55	93.47	91.91	92.17	88.51
**EfficientNetB1**	Accuracy(%)	86.68	92.95	92.43	93.47	88.77
	F1-Score(%)	86.61	92.86	92.43	93.39	88.63
	Precision(%)	86.87	93.24	92.65	93.79	88.95
	Recall(%)	86.68	92.95	92.43	93.47	88.77
**EfficientNetB2**	Accuracy(%)	84.33	86.95	92.17	92.43	89.56
	F1-Score(%)	84.18	86.87	92.17	92.32	89.39
	Precision(%)	84.64	87.33	92.34	92.59	89.94
	Recall(%)	84.33	86.95	92.17	92.43	89.56
**EfficientNetB3**	Accuracy(%)	86.16	92.43	92.95	93.47	90.08
	F1-Score(%)	86.03	92.43	92.97	93.31	89.97
	Precision(%)	86.25	92.57	93.08	93.78	90.45
	Recall(%)	86.16	92.43	92.95	93.47	90.08
**EfficientNetB4**	Accuracy(%)	87.73	91.38	93.21	93.21	91.64
	F1-Score(%)	87.66	91.32	93.25	93.2	91.52
	Precision(%)	87.9	91.9	93.62	93.37	91.89
	Recall(%)	87.73	91.38	93.21	93.21	91.64
**EfficientNetB5**	Accuracy(%)	84.6	91.91	91.64	92.43	89.82
	F1-Score(%)	84.39	91.83	91.63	92.26	89.68
	Precision(%)	84.89	92.17	91.79	93.0	90.41
	Recall(%)	84.6	91.91	91.64	92.43	89.82
**EfficientNetB6**	Accuracy(%)	84.6	86.68	92.17	91.12	85.64
	F1-Score(%)	84.38	86.69	92.2	90.66	85.59
	Precision(%)	84.64	87.22	92.31	91.79	85.9
	Recall(%)	84.6	86.68	92.17	91.12	85.64
**EfficientNetB7**	Accuracy(%)	83.29	89.82	90.86	90.6	89.3
	F1-Score(%)	83.12	89.64	90.88	90.05	89.31
	Precision(%)	83.5	90.26	91.1	90.94	89.87
	Recall(%)	83.29	89.82	90.86	90.6	89.3
**InceptionResNetV2**	Accuracy(%)	38.38	50.39	68.15	50.39	55.09
	F1-Score(%)	26.38	46.34	65.87	43.16	48.45
	Precision(%)	30.46	51.58	68.9	39.08	55.55
	Recall(%)	38.38	50.39	68.15	50.39	55.09
**InceptionV3**	Accuracy(%)	69.97	86.95	91.64	90.08	85.12
	F1-Score(%)	69.41	86.55	91.73	89.93	85.01
	Precision(%)	71.24	86.98	92.01	90.62	86.6
	Recall(%)	69.97	86.95	91.64	90.08	85.12
**MobileNet**	Accuracy(%)	75.2	93.99	91.38	90.08	79.9
	F1-Score(%)	75.1	93.98	91.45	89.97	79.55
	Precision(%)	76.79	94.1	91.65	90.41	80.59
	Recall(%)	75.2	93.99	91.38	90.08	79.9
**MobileNetV2**	Accuracy(%)	80.16	91.38	89.82	90.08	82.25
	F1-Score(%)	79.99	91.27	89.9	90.0	81.49
	Precision(%)	81.5	91.34	90.63	90.31	82.24
	Recall(%)	80.16	91.38	89.82	90.08	82.25
**MobileNetV3Large**	Accuracy(%)	83.03	94.26	93.21	93.21	89.82
	F1-Score(%)	82.75	94.25	93.2	93.19	89.67
	Precision(%)	83.51	94.51	93.32	93.57	89.85
	Recall(%)	83.03	94.26	93.21	93.21	89.82
**MobileNetV3Small**	Accuracy(%)	81.98	93.21	93.21	92.95	90.08
	F1-Score(%)	81.62	93.1	93.2	92.92	90.03
	Precision(%)	81.85	93.42	93.23	93.07	90.78
	Recall(%)	81.98	93.21	93.21	92.95	90.08
**Xception**	Accuracy(%)	70.23	89.3	91.38	90.86	82.25
	F1-Score(%)	68.59	89.27	91.32	90.87	82.02
	Precision(%)	68.47	89.54	91.61	91.1	83.31
	Recall(%)	70.23	89.3	91.38	90.86	82.25

**Table 6:** Security performance of models on AMD dataset.

Model	Metric	APK	AM	CD	DAM	SMALI
**Ours**	Accuracy(%)	89.02	93.94	95.15	97.49	92.09
	F1-Score(%)	88.69	93.77	95.17	97.48	92.19
	Precision(%)	88.81	94.18	95.25	97.51	93.0
	Recall(%)	89.02	93.94	95.15	97.49	92.09
**VGG16**	Accuracy(%)	83.86	92.24	93.45	91.02	90.64
	F1-Score(%)	83.23	92.22	93.36	90.93	90.41
	Precision(%)	83.42	92.2	93.37	91.14	90.66
	Recall(%)	83.86	92.24	93.45	91.02	90.64
**ResNet50**	Accuracy(%)	85.96	92.72	94.66	93.77	91.53
	F1-Score(%)	85.58	92.61	94.64	93.61	91.48
	Precision(%)	85.89	92.66	94.66	93.77	91.46
	Recall(%)	85.96	92.72	94.66	93.77	91.53
**VGG19**	Accuracy(%)	85.79	92.81	93.78	90.21	89.99
	F1-Score(%)	85.35	92.7	93.78	90.12	89.66
	Precision(%)	85.5	92.69	93.8	90.14	89.67
	Recall(%)	85.79	92.81	93.78	90.21	89.99
**DenseNet121**	Accuracy(%)	80.71	90.78	93.45	91.59	89.1
	F1-Score(%)	80.14	90.48	93.4	91.4	89.08
	Precision(%)	80.64	90.56	93.45	91.37	89.35
	Recall(%)	80.71	90.78	93.45	91.59	89.1
**DenseNet169**	Accuracy(%)	81.03	90.38	93.45	90.53	89.43
	F1-Score(%)	80.21	89.78	93.34	90.27	89.36
	Precision(%)	80.69	90.05	93.41	90.25	89.39
	Recall(%)	81.03	90.38	93.45	90.53	89.43
**DenseNet201**	Accuracy(%)	84.99	88.44	93.13	90.78	90.15
	F1-Score(%)	84.56	87.55	93.05	90.6	90.1
	Precision(%)	84.55	88.51	93.08	90.58	90.1
	Recall(%)	84.99	88.44	93.13	90.78	90.15
**EfficientNetB0**	Accuracy(%)	85.96	90.7	93.61	87.86	88.86
	F1-Score(%)	85.63	90.32	93.55	86.74	88.48
	Precision(%)	85.94	90.37	93.56	88.24	88.53
	Recall(%)	85.96	90.7	93.61	87.86	88.86
**EfficientNetB1**	Accuracy(%)	87.17	91.35	93.53	89.16	89.18
	F1-Score(%)	86.86	91.11	93.54	88.51	89.31
	Precision(%)	87.1	91.09	93.56	88.97	89.95
	Recall(%)	87.17	91.35	93.53	89.16	89.18
**EfficientNetB2**	Accuracy(%)	84.5	89.98	92.64	91.1	88.78
	F1-Score(%)	83.98	89.76	92.59	90.7	88.47
	Precision(%)	84.09	89.76	92.62	90.88	89.0
	Recall(%)	84.5	89.98	92.64	91.1	88.78
**EfficientNetB3**	Accuracy(%)	85.39	91.27	93.45	91.83	88.86
	F1-Score(%)	84.59	90.98	93.47	91.61	88.71
	Precision(%)	85.41	91.06	93.51	91.64	88.66
	Recall(%)	85.39	91.27	93.45	91.83	88.86
**EfficientNetB4**	Accuracy(%)	87.81	89.17	93.37	91.02	89.91
	F1-Score(%)	87.45	88.47	93.29	90.62	89.88
	Precision(%)	87.55	88.99	93.34	90.75	90.07
	Recall(%)	87.81	89.17	93.37	91.02	89.91
**EfficientNetB5**	Accuracy(%)	85.55	89.25	93.86	90.13	89.1
	F1-Score(%)	84.72	88.79	93.79	89.89	88.89
	Precision(%)	85.45	88.84	93.78	89.94	88.83
	Recall(%)	85.55	89.25	93.86	90.13	89.1
**EfficientNetB6**	Accuracy(%)	87.25	88.12	92.89	91.18	86.76
	F1-Score(%)	86.65	87.74	92.83	91.22	86.73
	Precision(%)	87.46	87.66	92.82	91.33	87.67
	Recall(%)	87.25	88.12	92.89	91.18	86.76
**EfficientNetB7**	Accuracy(%)	86.6	91.59	93.69	91.75	88.62
	F1-Score(%)	85.96	91.34	93.62	91.46	88.47
	Precision(%)	86.29	91.33	93.67	91.67	88.58
	Recall(%)	86.6	91.59	93.69	91.75	88.62
**InceptionResNetV2**	Accuracy(%)	47.78	64.51	71.95	47.09	54.8
	F1-Score(%)	36.97	58.54	72.15	40.7	46.95
	Precision(%)	54.67	58.44	75.97	50.22	44.31
	Recall(%)	47.78	64.51	71.95	47.09	54.8
**InceptionV3**	Accuracy(%)	65.86	86.5	92.64	89.08	81.68
	F1-Score(%)	64.39	85.98	92.57	88.59	80.69
	Precision(%)	73.89	86.16	92.67	88.55	80.74
	Recall(%)	65.86	86.5	92.64	89.08	81.68
**MobileNet**	Accuracy(%)	79.58	90.22	91.35	88.92	82.41
	F1-Score(%)	78.27	90.12	91.33	88.17	81.64
	Precision(%)	78.52	90.07	91.37	88.71	81.7
	Recall(%)	79.58	90.22	91.35	88.92	82.41
**MobileNetV2**	Accuracy(%)	79.58	89.01	90.22	89.16	82.0
	F1-Score(%)	78.59	88.57	90.25	88.81	81.97
	Precision(%)	78.57	88.85	90.51	88.78	83.08
	Recall(%)	79.58	89.01	90.22	89.16	82.0
**MobileNetV3Large**	Accuracy(%)	85.39	91.03	93.53	92.72	90.4
	F1-Score(%)	85.04	90.97	93.56	92.44	90.38
	Precision(%)	85.04	90.94	93.62	92.74	90.41
	Recall(%)	85.39	91.03	93.53	92.72	90.4
**MobileNetV3Small**	Accuracy(%)	85.15	91.11	93.69	91.67	89.91
	F1-Score(%)	84.77	91.03	93.69	91.61	89.79
	Precision(%)	85.01	91.0	93.75	91.59	89.87
	Recall(%)	85.15	91.11	93.69	91.67	89.91
**Xception**	Accuracy(%)	76.27	89.57	90.22	90.29	83.29
	F1-Score(%)	75.52	89.24	90.28	90.04	82.96
	Precision(%)	75.85	89.45	90.38	89.95	83.0
	Recall(%)	76.27	89.57	90.22	90.29	83.29

Tables 5 and 6 present the performance of all predictive models generated based on the DREBIN and AMD datasets from security perspectives. The results revealed that the proposed CNN algorithm achieves superior detection efficacy for the assessed security parameters compared to the other conventional CNN algorithms. Furthermore, it is demonstrated for the two examined android malware datasets that the DAM vision-based format introduces the best security performance for the proposed CNN algorithm and almost all tested CNN algorithms compared to other examined vision-based formats.

Moreover, Tables 5 and 6 show that the achievement of high detection efficacy depends on the proper selection of the CNN algorithm and the appropriate choice of utilized vision-based format. So, for example, in some tested cases, the DAM vision-based format is not the best vision-based scenario for some examined CNN algorithms. Therefore, based on the security target of the android malware analysis system, it can select the appropriate CNN model and vision-based format.

**Fig 4:**
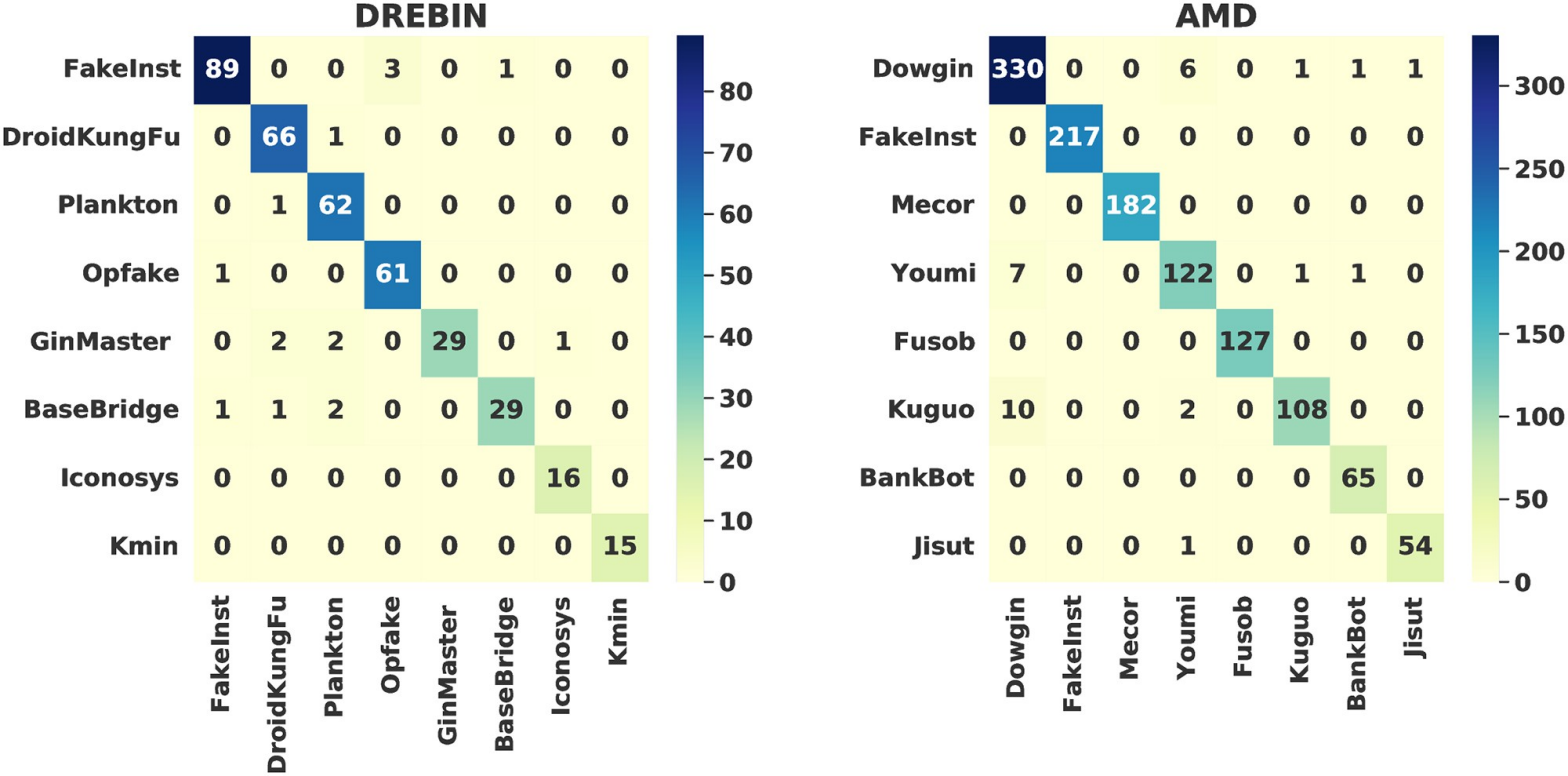
Confusion matrix of the proposed CNN algorithm (DAM format).

**Fig 5:**
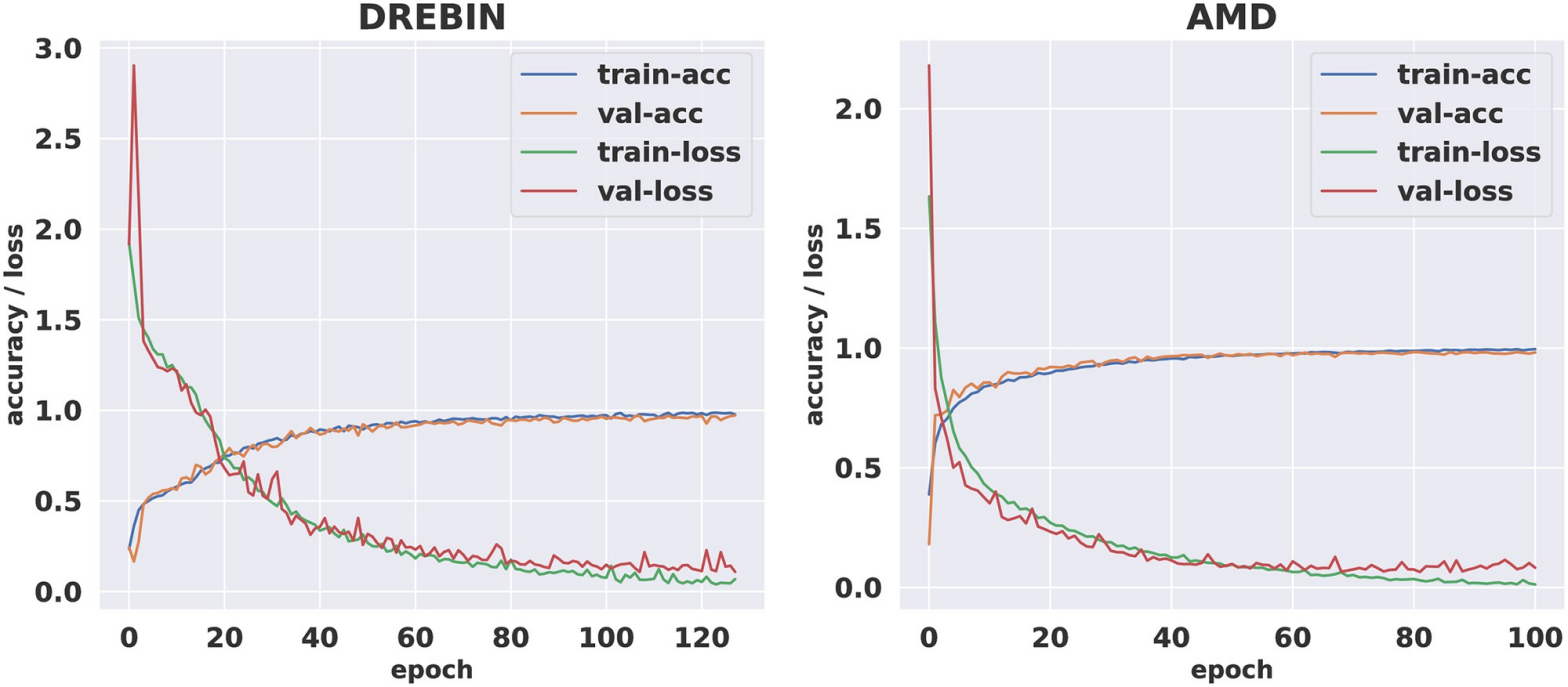
DREBIN VS AMD in terms of best model acc-loss chart.

22 CNN models were implemented and applied on the two datasets. To simplify the presentation of the simulation results, we introduce only the confusion matrices and the accuracy & loss curves of the best-performed CNN model for the two investigated android malware datasets. Fig 4 presents the acquired confusion matrices of the proposed CNN algorithm for the two tested AMD and DREBIN android malware datasets for the best DAM image format. The security performance evaluation in terms of accuracy, recall, precision, and F1-Score can be estimated from these confusion matrices. It is demonstrated that the proposed CNN algorithm gives low false detection and low misclassification rate for the eight examined malware classes in both datasets. Fig 5 introduces the obtained accuracy & loss curves of the proposed CNN algorithm for the two tested AMD and DREBIN android malware datasets for the best DAM image format. The achieved results confirm that the proposed CNN algorithm provides the highest detection accuracy and the lowest detection loss compared to the other examined CNN algorithms, as also clarified in Tables 5 and 6.

Table 7 shows the highest increase in the performance achieved among the different predictive models in terms of accuracy, F1-Score, precision, and recall. The comparison was conducted to show how various factors can affect the performance of the resulting predictive models in case of (a) only changing the type of conversion while keeping the same dataset and the applied CNN algorithm, (b) keeping the same conversion type and dataset while changing the applied CNN algorithm (c) keeping the type of conversion and applied CNN algorithm while changing the dataset itself. For example, the accuracy improvement reached 52.80% when CD type is used instead of the whole APK utilizing InceptionResNetV2 algorithm and AMD dataset. On the other hand, the accuracy improved by 107% when DAM type was used by our proposed algorithm (scratch) in comparison to the InceptionResNetV2 algorithm. The rest of the most significant F1-score, precision, and recall improvements have reached 95.16%, 71.44%, and 52.8%, respectively, when different conversion types were considered while applying the same CNN algorithm. Additionally, within the same conversion type, applying different CNN algorithms introduced 139.91% of F1-score improvement in the case of DAM type, 109.88% precision improvement in the case of SMALI type, and 107.04% in the case of DAM type.

**Table 7:** Comparative analysis: Percentage of the highest improved performance among different CNN models per metric and dataset type.

Comparative Analysis Metrics	Accuracy(%)	F1-Score(%)	Precision(%)	Recall(%)
AMD-Conversions	52.80	95.16	71.44	52.80
AMD-Algorithms	107.04	139.91	109.88	107.04
DERBIN-Conversions	77.55	149.71	126.16	77.55
DERBIN-Algorithms	131.29	236.44	192.00	131.29

Similar behaviors were observed when DREBIN dataset was used. When applying the same CNN algorithm but considering different conversion types, the accuracy, F1-Score, precision, and recall have been improved by 77.55%, 149.71%, 126.16%, and 77.55%, respectively. However, the highest increase in the performance reached 131.29%, 236.44%, 192.00%, and 131.29% in terms of accuracy, F1-Score, precision and recall, respectively, when APK format was used and different CNN algorithms were applied.

The performance was also affected when the dataset itself was changed. For example, the amount of improvement in the accuracy, F1-Score, precision, and recall was higher when the DREBIN dataset was used, whether by changing the conversion type or the applied CNN algorithm, as also shown in Table 7.

About the above comparisons and discussion, we can emphasize the impact of different factors on the performance of the android malware analysis systems. These factors need to be carefully addressed by the developers of the malware detection system to build predictive models that meet their needs.

In the following section, another way of assessing the malware analysis systems in terms of complexity. The developers can balance both the security and complexity measures when building their systems.

### Complexity analysis

In addition to the security evaluation of the proposed comprehensive android malware analysis and predictive model, we have measured the complexity concerning the models’ execution and pre-processing phases. The models’ execution cost was calculated based on the model computational test time and CPU usage. Therefore, the complexity of all examined CNN algorithms, including our proposed algorithm, was measured when the two different android malware datasets were utilized, as shown in Tables 8 and 9. The experiments’ outcomes reveal that (a) in the case of the DREBIN dataset, the test time was higher when APK as a whole was converted to an image, especially in our proposed CNN algorithm, VGG16, ResNet50, DenseNet121, DenseNet169, and EfficientNetB0, (b) there was variation among the models in regards to testing time even after using the same conversion type, (c) CPU usage in case of APK was less or close to the other types’ CPU usages in almost all CNN algorithms. In general, the CPU usage values were close in all algorithms for all conversion types, (d) overall, the test time was higher in DREBIN in comparison to AMD in all applied CNN algorithms, (e) fewer variations among the test time in the case of using AMD dataset in comparison to DREBIN with the highest value observed in EfficientNetB7, (f) CPU usage values were close for all conversion types and applied CNN algorithms in the case of using the AMD dataset, (g) our proposed CNN algorithm achieved lower testing time and CPU usage compared to other transfer learning CNN algorithms for the two tested datasets.

**Table 8:** Complexity performance of models on DREBIN dataset.

Model	Metric	APK	AM	CD	DAM	SMALI
**Ours**	Avg. Test Time(ms)	6.43	0.53	0.54	0.54	0.54
	Avg. CPU Usage(GB)	0.0094	0.0104	0.0107	0.0113	0.0113
**VGG16**	Avg. Test Time(ms)	11.98	1.54	1.45	1.45	1.47
	Avg. CPU Usage(GB)	0.0096	0.0105	0.0108	0.0115	0.0115
**ResNet50**	Avg. Test Time(ms)	4.07	2.79	2.67	4.18	2.68
	Avg. CPU Usage(GB)	0.0096	0.0107	0.011	0.0109	0.0116
**VGG19**	Avg. Test Time(ms)	1.66	1.67	1.67	1.66	1.68
	Avg. CPU Usage(GB)	0.0097	0.0108	0.0111	0.0109	0.0118
**DenseNet121**	Avg. Test Time(ms)	7.74	4.39	4.3	4.24	4.23
	Avg. CPU Usage(GB)	0.0097	0.0104	0.0106	0.0111	0.0112
**DenseNet169**	Avg. Test Time(ms)	7.15	5.67	5.67	5.79	5.78
	Avg. CPU Usage(GB)	0.0099	0.0104	0.0106	0.0109	0.0114
**DenseNet201**	Avg. Test Time(ms)	8.5	7.91	6.99	6.96	7.0
	Avg. CPU Usage(GB)	0.0101	0.0105	0.0107	0.011	0.0113
**EfficientNetB0**	Avg. Test Time(ms)	5.29	3.18	5.01	3.2	3.22
	Avg. CPU Usage(GB)	0.01	0.0103	0.0107	0.011	0.0115
**EfficientNetB1**	Avg. Test Time(ms)	4.76	5.77	4.44	4.48	4.54
	Avg. CPU Usage(GB)	0.0099	0.0104	0.0107	0.0111	0.0114
**EfficientNetB2**	Avg. Test Time(ms)	5.62	5.28	4.5	6.08	4.5
	Avg. CPU Usage(GB)	0.01	0.0104	0.0108	0.011	0.0115
**EfficientNetB3**	Avg. Test Time(ms)	7.58	5.13	5.23	5.22	5.32
	Avg. CPU Usage(GB)	0.01	0.0104	0.0107	0.011	0.0114
**EfficientNetB4**	Avg. Test Time(ms)	8.88	6.46	6.6	6.55	6.47
	Avg. CPU Usage(GB)	0.01	0.0105	0.0107	0.0111	0.0114
**EfficientNetB5**	Avg. Test Time(ms)	9.78	8.02	8.09	8.12	8.13
	Avg. CPU Usage(GB)	0.0101	0.0105	0.0108	0.011	0.0115
**EfficientNetB6**	Avg. Test Time(ms)	11.73	10.87	9.64	9.65	9.66
	Avg. CPU Usage(GB)	0.0103	0.0105	0.0109	0.0111	0.0114
**EfficientNetB7**	Avg. Test Time(ms)	15.02	13.73	12.15	12.17	12.14
	Avg. CPU Usage(GB)	0.0104	0.0106	0.011	0.0113	0.0115
**InceptionResNetV2**	Avg. Test Time(ms)	9.88	9.08	7.61	7.75	7.9
	Avg. CPU Usage(GB)	0.0106	0.0107	0.0111	0.0114	0.0116
**InceptionV3**	Avg. Test Time(ms)	6.87	4.43	3.14	3.14	3.1
	Avg. CPU Usage(GB)	0.0103	0.0106	0.0111	0.0116	0.0118
**MobileNet**	Avg. Test Time(ms)	1.92	1.29	1.3	4.16	1.28
	Avg. CPU Usage(GB)	0.0105	0.0107	0.0113	0.0114	0.0119
**MobileNetV2**	Avg. Test Time(ms)	2.37	1.91	1.93	1.98	1.92
	Avg. CPU Usage(GB)	0.0104	0.0108	0.0114	0.0113	0.0121
**MobileNetV3Large**	Avg. Test Time(ms)	3.52	3.67	4.56	2.53	2.54
	Avg. CPU Usage(GB)	0.0105	0.0106	0.011	0.0115	0.0117
**MobileNetV3Small**	Avg. Test Time(ms)	3.79	2.02	2.08	2.05	2.11
	Avg. CPU Usage(GB)	0.0104	0.0107	0.011	0.0116	0.0118
**Xception**	Avg. Test Time(ms)	4.41	3.61	2.55	2.53	2.53
	Avg. CPU Usage(GB)	0.0105	0.0106	0.0112	0.0112	0.0119

**Table 9:** Complexity performance of models on AMD dataset.

Model	Metric	APK	AM	CD	DAM	SMALI
**Ours**	Avg. Test Time(ms)	0.42	0.4	0.31	0.41	0.31
	Avg. CPU Usage(GB)	0.0039	0.004	0.0045	0.0045	0.0049
**VGG16**	Avg. Test Time(ms)	2.44	2.44	1.19	2.4	1.19
	Avg. CPU Usage(GB)	0.004	0.0041	0.0046	0.0047	0.0051
**ResNet50**	Avg. Test Time(ms)	1.73	1.74	1.52	1.73	1.57
	Avg. CPU Usage(GB)	0.0042	0.0042	0.0048	0.0048	0.0052
**VGG19**	Avg. Test Time(ms)	1.38	1.39	1.4	1.39	1.39
	Avg. CPU Usage(GB)	0.0043	0.0044	0.0049	0.0049	0.0054
**DenseNet121**	Avg. Test Time(ms)	2.63	2.7	2.15	3.82	2.16
	Avg. CPU Usage(GB)	0.0038	0.0045	0.0039	0.004	0.0041
**DenseNet169**	Avg. Test Time(ms)	3.04	3.88	2.82	3.05	2.86
	Avg. CPU Usage(GB)	0.0038	0.0038	0.0041	0.0041	0.0042
**DenseNet201**	Avg. Test Time(ms)	3.67	3.66	4.36	3.7	3.46
	Avg. CPU Usage(GB)	0.0039	0.004	0.0039	0.004	0.0041
**EfficientNetB0**	Avg. Test Time(ms)	1.81	1.85	1.64	1.87	1.68
	Avg. CPU Usage(GB)	0.0037	0.0038	0.004	0.0041	0.0042
**EfficientNetB1**	Avg. Test Time(ms)	2.31	2.28	2.22	2.29	2.25
	Avg. CPU Usage(GB)	0.0039	0.004	0.0039	0.0042	0.0043
**EfficientNetB2**	Avg. Test Time(ms)	2.45	2.44	2.3	2.43	2.26
	Avg. CPU Usage(GB)	0.004	0.0038	0.004	0.0044	0.0045
**EfficientNetB3**	Avg. Test Time(ms)	3.01	3.0	2.73	3.02	4.01
	Avg. CPU Usage(GB)	0.0038	0.004	0.0042	0.004	0.0041
**EfficientNetB4**	Avg. Test Time(ms)	3.64	3.66	4.46	3.68	3.47
	Avg. CPU Usage(GB)	0.0038	0.0041	0.0039	0.0041	0.0042
**EfficientNetB5**	Avg. Test Time(ms)	4.65	4.65	4.37	5.8	4.38
	Avg. CPU Usage(GB)	0.0039	0.0038	0.004	0.004	0.0041
**EfficientNetB6**	Avg. Test Time(ms)	5.63	6.63	5.36	5.69	5.36
	Avg. CPU Usage(GB)	0.0038	0.0038	0.0039	0.0041	0.0042
**EfficientNetB7**	Avg. Test Time(ms)	8.06	7.08	6.79	7.16	6.8
	Avg. CPU Usage(GB)	0.0038	0.0039	0.004	0.004	0.0041
**InceptionResNetV2**	Avg. Test Time(ms)	4.18	4.22	3.89	4.17	3.92
	Avg. CPU Usage(GB)	0.0038	0.004	0.0042	0.0042	0.0043
**InceptionV3**	Avg. Test Time(ms)	1.89	1.85	1.56	1.89	1.57
	Avg. CPU Usage(GB)	0.0039	0.0042	0.0043	0.0041	0.0044
**MobileNet**	Avg. Test Time(ms)	0.79	0.78	0.7	0.79	0.71
	Avg. CPU Usage(GB)	0.0041	0.0043	0.0044	0.0042	0.0045
**MobileNetV2**	Avg. Test Time(ms)	1.05	1.06	2.22	1.06	0.98
	Avg. CPU Usage(GB)	0.0042	0.0039	0.004	0.0044	0.0047
**MobileNetV3Large**	Avg. Test Time(ms)	1.35	1.37	1.21	1.36	2.64
	Avg. CPU Usage(GB)	0.0044	0.004	0.0041	0.0045	0.0041
**MobileNetV3Small**	Avg. Test Time(ms)	1.05	1.07	0.93	1.06	0.94
	Avg. CPU Usage(GB)	0.0045	0.0042	0.0042	0.0047	0.0042
**Xception**	Avg. Test Time(ms)	2.84	1.91	1.58	1.9	1.59
	Avg. CPU Usage(GB)	0.0038	0.0043	0.0044	0.0048	0.0044

As discussed in the propose work section, the CNN algorithm is developed from scratch, and it is not a pre-trained CNN algorithm. Thus, as clarified in Table 2 (last row), our proposed CNN algorithm used a small number of trainable/non-trainable parameters compared to other pre-trained CNN algorithms. Therefore, it introduced a lower execution time.

**Fig 6:**
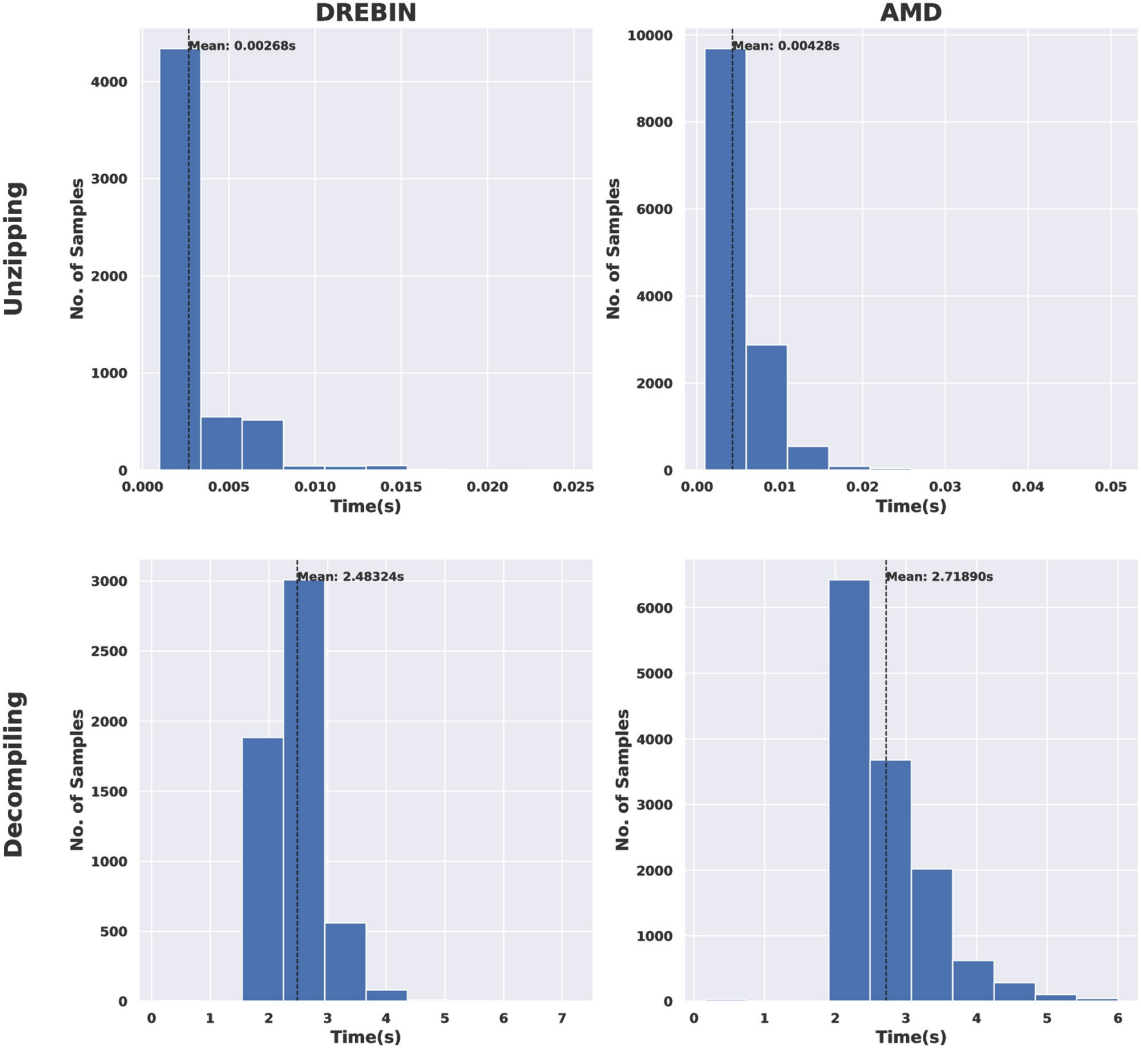
DREBIN VS AMD in terms of speed of decompiling and unzipping processes.

Furthermore, the complexity of the pre-processing phases was measured in terms of (a) the speed of decompiling and unzipping processes for both the two tested android malware datasets, and (b) the size of the obtained visual images for all types of conversion considered in this research. Fig 6 demonstrates, in terms of histograms, the speed of decompiling and unzipping processes for the DREBIN and AMD datasets.

The histogram distributions show the number of samples (y-axis) unzipped or decompiled as the time elapsed (x-axis). The general observation is that the unzipping process is faster than the decompiling for the two examined android malware datasets. This can be witnessed by counting the number of samples that can be unzipped by time. For example, in the case of the DREBIN dataset, more than 4000 apps took less than 0.005 seconds to be unzipped. In contrast, most apps (around 3000) took 2 to 3 seconds to be decompiled. AMD dataset apps took less time to unzip and decompile. For example, around 10000 apps took less than 0.01 seconds to be unzipped. In comparison, about 7000 apps took less than 3 seconds to be decompiled.

Similar outcomes are observed in the case of using the AMD dataset. However, overall, the unzipping and decompilation processes were faster in DREBIN than in the AMD dataset; this is due to the nature of the android apps included in this dataset.

**Fig 7:**
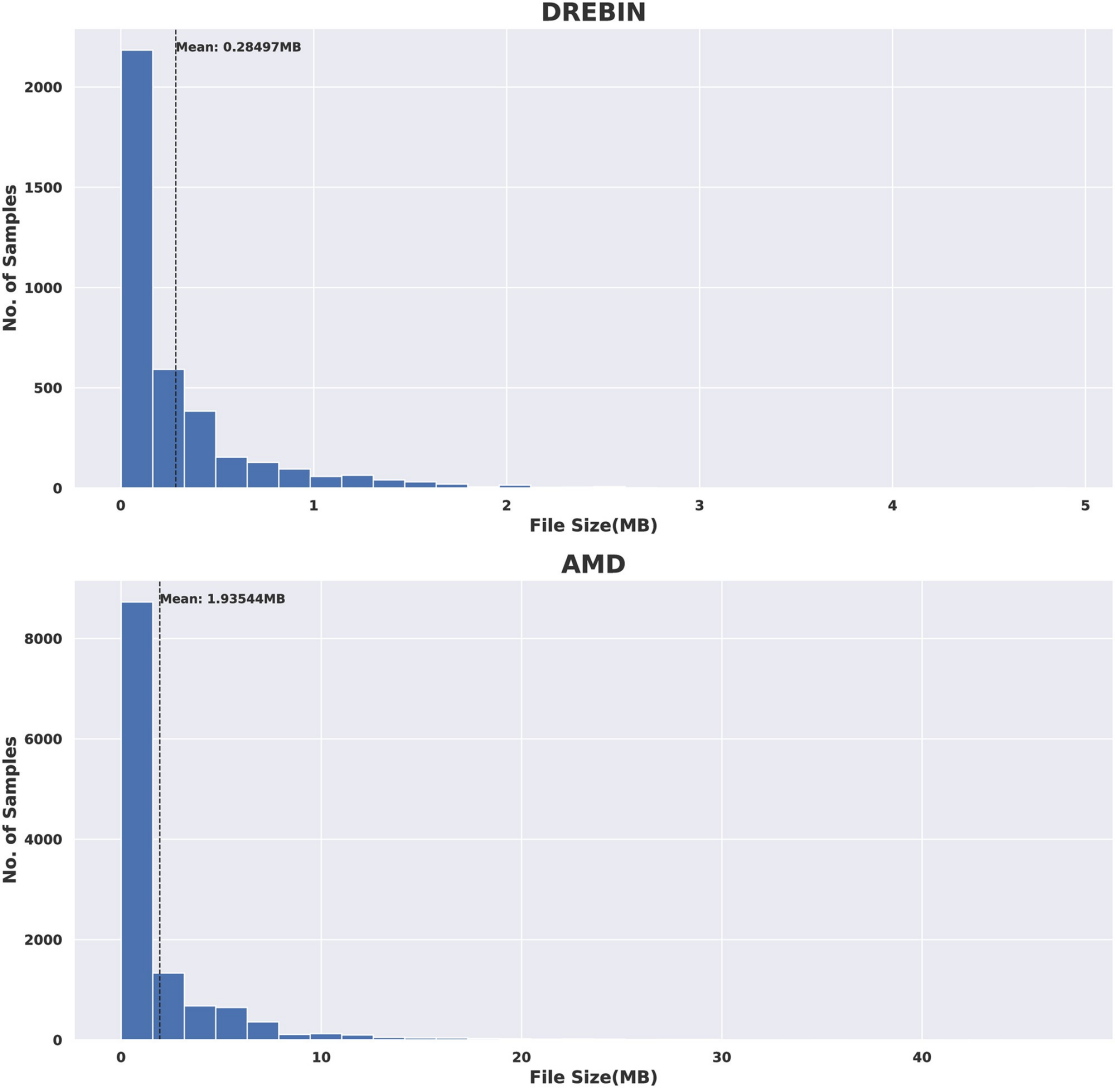
DREBIN VS AMD in terms of file size of APK images.

**Fig 8:**
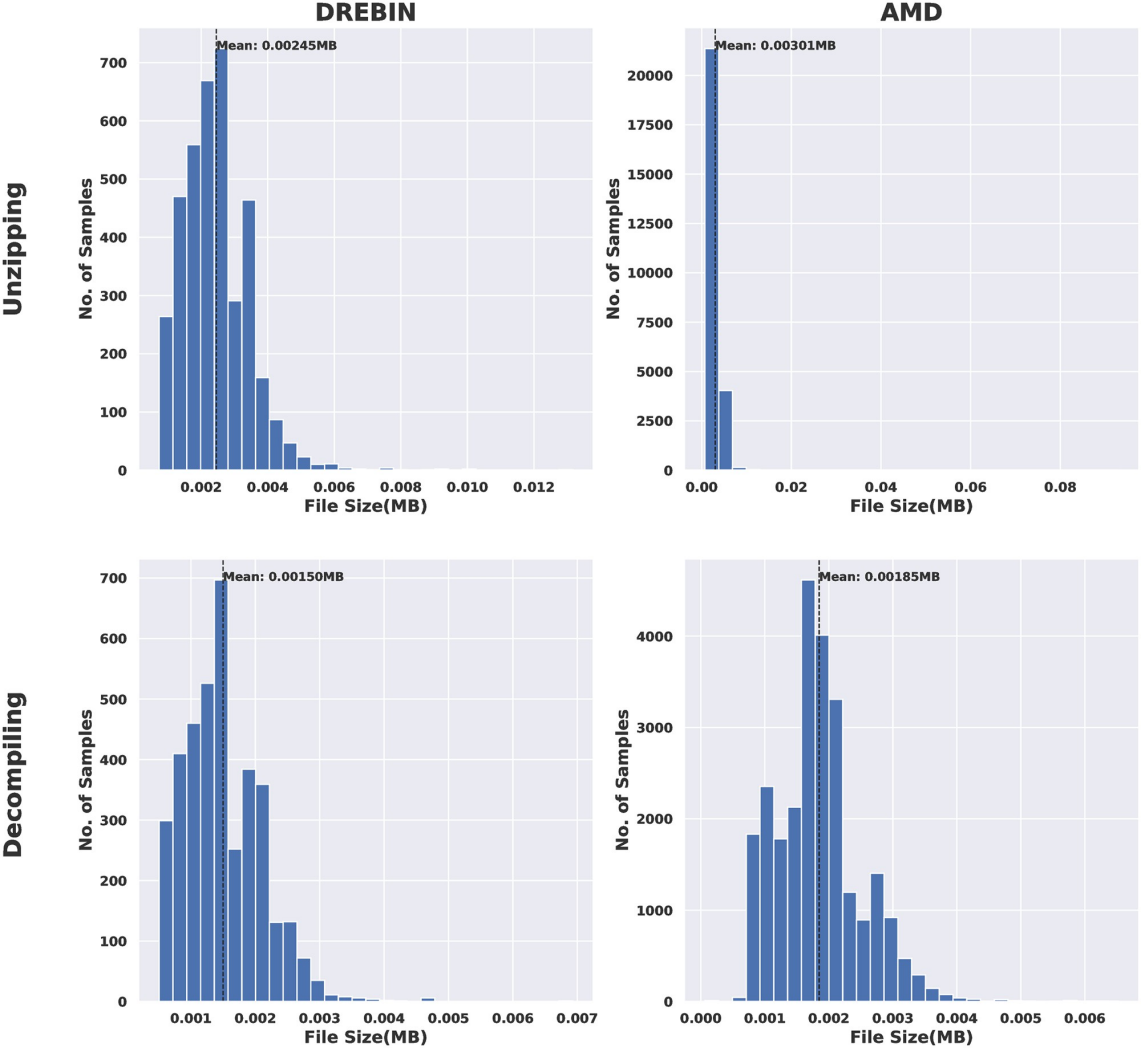
DREBIN VS AMD in terms of file size of AM/DAM images.

**Fig 9:**
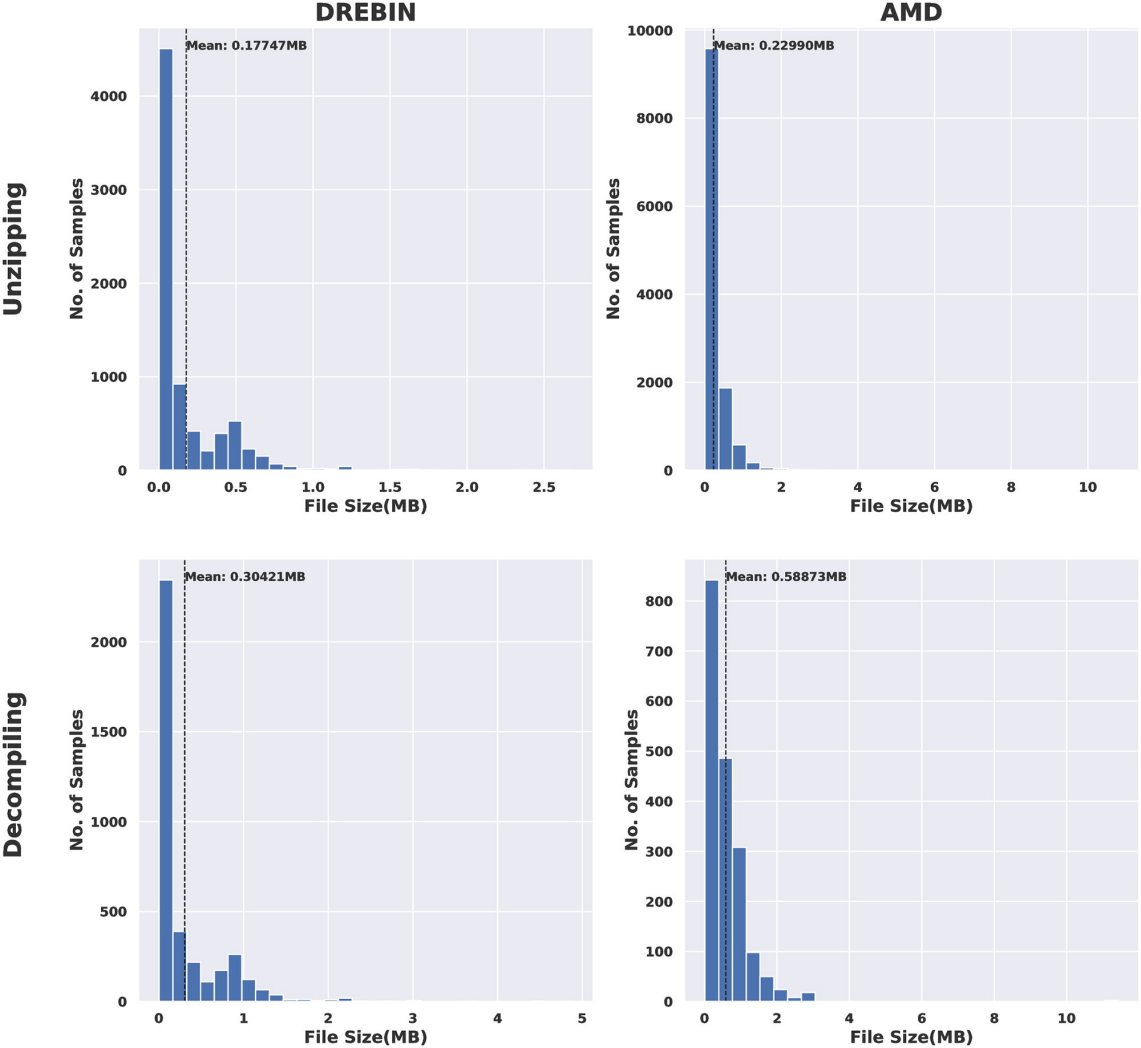
DREBIN VS AMD in terms of file size of CD/SMALI images.

Moreover, Figs 7–9 show the histogram distribution of the files size of the resulted images from the different types of conversations for the two datasets. Fig 7 presents the files size comparison between the DREBIN and AMD datasets of the produced APK images. It can be noticed that files size was much larger when the AMD dataset was used.

The files size comparisons between the DREBIN and AMD datasets in the case of AM/DAM images and CD/SMALI images are shown in Figs 8 and 9, respectively. The obtained results declare that the APK images have the largest files size compared to the other images resulting from other files types for both datasets. However, AM/DAM images are the smallest among them. Moreover, for all types of files and produced images, AMD was higher in size than DREBIN. Again this is due to the nature of the android apps included in this dataset.

## Conclusions and future work

Android is the leading operating system worldwide, with around 70% market share. Consequently, attracting different security attackers to produce threatening malware apps that serve their bad intentions. On the other hand, security professionals are highly motivated to build efficient and smart android malware analysis and detection systems. These systems could be built based on vision-based approaches where the android apps or some of their components are converted to images. In this context, CNN algorithms are one of the best choices to generate vision-based predictive solutions.

The main shortcoming of the current related works is the focus on some factors when developing their malware analysis solutions, limiting the selection of best factors and practices that meet the target performance within the available resources.

Therefore, this study aims to provide a nutshell model for analyzing android malware apps that facilitates achieving high performance while respecting the system’s constraints. Furthermore, this research studied intensely the main factors that might significantly influence the performance of detecting android malware from security and complexity perspectives.

This study started by conducting a deep comparison among recent related works in the area of vision-based android malware analysis to check the primary factors considered by them and their ways of assessing them. Then we have built a comprehensive malware analysis model that captures essential aspects, processes, and practices that need to be considered to ensure the efficient building of malware detection systems. This model provides a thorough vision to developers on what to choose and why based on the systems’ needs and resources.

The primary factors that are included in our proposed model are: the type of conversions that decide on which features will be converted to images and how, dataset nature that depends on the kind of android malware apps included in the dataset, CNN algorithms that will be used to build the malware predictive solution, and most importantly the evaluation process that comprehensively assesses the performance of the malware analysis system in terms of complexity and security.

A deep empirical study has been conducted to evaluate the proposed model. The results reveal that the chosen factors and processes can significantly impact the performance of the analysis model, whether in terms of the security metrics such as accuracy, F1-score, precision, recall, or the complexity metrics such as test time, CPU usage, storage size, and pre-processing speed.

As a result, the proposed model will effectively direct the developers of malware analysis systems on which factors to adopt based on their requirements and the chosen factors’ impacts. Therefore, the researchers and developers can benefit from our model to trade off these factors to ensure building malware analysis systems that meet their goals.

For future work, other comprehensive models could be proposed for android malware analysis systems that are not vision-based. Additionally, we could introduce nutshell analysis models for different types of malware to other kinds of operating systems. Furthermore, we intend to study the effect of using variable byte sizes and different image sizes for the visual features of the Android malware applications. Moreover, a deep analysis of different misclassification and obfuscation classification scenarios can be investigated.

## Appendix A

S1 and S2 Tables illustrate the security performance of different CNN algorithms utilizing DREBIN and AMD datasets, respectively. As mentioned before, these metrics were not included in the analysis section for simplicity in presenting the results and highlighting the main evaluation metrics in regards to the detection performance.

## Supporting information

S1 Table Security performance of models on DREBIN dataset based on other metrics. (PDF)

S2 Table Security performance of models on AMD dataset based on other metrics. (PDF)
