# Supplementary material for: Android malware analysis in a nutshell
Source: PLoS One. 2022 Jul 5;17(7):e0270647. doi: 10.1371/journal.pone.0270647 (PMC9255778; doi:10.1371/journal.pone.0270647)
Supplement: S1 Table — (PDF) [file pone.0270647.s001.pdf]

Table 10. Security performance of models on DREBIN dataset based on other metrics

Model	Format	TNR	NPV	FPR	FNR	FDR	TPR	FOR	MR
**Scratch**	APK	98.35	98.37	1.65	14.62	11.73	85.38	1.63	2.81
	AM	99.09	99.08	0.91	6.83	7.4	93.17	0.92	1.57
	CD	99.2	99.2	0.8	5.55	6.11	94.45	0.8	1.37
	DAM	99.37	99.39	0.63	4.48	3.66	95.52	0.61	1.04
	SMALI	98.67	98.66	1.33	9.27	9.54	90.73	1.34	2.28
**VGG16**	APK	97.67	97.73	2.33	20.35	13.6	79.65	2.27	3.92
	AM	99.11	99.16	0.89	9.04	6.43	90.96	0.84	1.5
	CD	99.03	99.01	0.97	6.87	8.1	93.13	0.99	1.7
	DAM	98.95	99.0	1.05	8.58	7.05	91.42	1.0	1.76
	SMALI	98.34	98.47	1.66	16.84	10.79	83.16	1.53	2.74
**ResNet50**	APK	98.02	98.07	1.98	16.07	14.57	83.93	1.93	3.33
	AM	99.22	99.26	0.78	6.7	4.7	93.3	0.74	1.31
	CD	98.94	98.94	1.06	8.27	8.43	91.73	1.06	1.83
	DAM	99.33	99.38	0.67	5.95	4.49	94.05	0.62	1.11
	SMALI	98.62	98.63	1.38	10.59	9.65	89.41	1.37	2.35
**VGG19**	APK	97.57	97.61	2.43	20.54	16.77	79.46	2.39	4.11
	AM	99.12	99.14	0.88	6.84	5.56	93.16	0.86	1.5
	CD	99.21	99.19	0.79	5.56	6.4	94.44	0.81	1.37
	DAM	98.96	99.0	1.04	8.72	6.87	91.28	1.0	1.76
	SMALI	98.51	98.54	1.49	12.07	12.0	87.93	1.46	2.55
**DenseNet121**	APK	96.94	97.03	3.06	27.04	18.9	72.96	2.97	5.16
	AM	98.53	98.53	1.47	12.13	11.57	87.87	1.47	2.55
	CD	99.04	99.01	0.96	7.02	9.13	92.98	0.99	1.7
	DAM	98.99	99.05	1.01	8.92	5.98	91.08	0.95	1.7
	SMALI	97.88	97.95	2.12	20.31	14.79	79.69	2.05	3.59
**DenseNet169**	APK	97.62	97.69	2.38	19.92	14.8	80.08	2.31	3.98
	AM	98.75	98.76	1.25	9.86	10.22	90.14	1.24	2.15
	CD	98.85	98.82	1.15	8.09	9.14	91.91	1.18	2.02
	DAM	98.71	98.77	1.29	10.9	8.22	89.1	1.23	2.15
	SMALI	98.13	98.22	1.87	17.34	12.8	82.66	1.78	3.13
**DenseNet201**	APK	97.66	97.75	2.34	22.42	14.27	77.58	2.25	3.92
	AM	98.24	98.35	1.76	15.44	11.52	84.56	1.65	2.94
	CD	98.94	98.93	1.06	7.97	7.81	92.03	1.07	1.83
	DAM	98.92	98.96	1.08	9.16	7.82	90.84	1.04	1.83
	SMALI	98.34	98.36	1.66	12.44	11.26	87.56	1.64	2.81
**EfficientNetB0**	APK	97.58	97.61	2.42	20.29	17.16	79.71	2.39	4.11
	AM	99.03	99.06	0.97	7.8	5.53	92.2	0.94	1.63
	CD	98.82	98.81	1.18	7.73	8.21	92.27	1.19	2.02
	DAM	98.82	98.88	1.18	9.9	6.48	90.1	1.12	1.96
	SMALI	98.31	98.37	1.69	16.17	12.02	83.83	1.63	2.87
**EfficientNetB1**	APK	98.02	98.06	1.98	15.29	13.14	84.71	1.94	3.33
	AM	98.95	99.01	1.05	10.07	6.57	89.93	0.99	1.76
	CD	98.9	98.89	1.1	7.68	8.09	92.32	1.11	1.89
	DAM	99.02	99.09	0.98	9.3	6.03	90.7	0.91	1.63
	SMALI	98.33	98.4	1.67	15.31	11.65	84.69	1.6	2.81
**EfficientNetB2**	APK	97.67	97.72	2.33	18.69	16.65	81.31	2.28	3.92
	AM	98.08	98.14	1.92	18.82	14.66	81.18	1.86	3.26
	CD	98.87	98.86	1.13	8.17	9.64	91.83	1.14	1.96
	DAM	98.87	98.93	1.13	10.07	7.86	89.93	1.07	1.89
	SMALI	98.47	98.5	1.53	12.63	11.34	87.37	1.5	2.61

Model	Format	TNR	NPV	FPR	FNR	FDR	TPR	FOR	MR
**EfficientNetB3**	APK	97.95	97.99	2.05	17.0	14.32	83.0	2.01	3.46
	AM	98.89	98.9	1.11	8.82	7.62	91.18	1.1	1.89
	CD	98.97	98.98	1.03	7.84	8.05	92.16	1.02	1.76
	DAM	99.03	99.09	0.97	8.23	6.05	91.77	0.91	1.63
	SMALI	98.52	98.58	1.48	12.41	9.51	87.59	1.42	2.48
**EfficientNetB4**	APK	98.21	98.22	1.79	14.81	13.89	85.19	1.78	3.07
	AM	98.7	98.77	1.3	10.73	6.69	89.27	1.23	2.15
	CD	99.0	99.02	1.0	6.91	6.58	93.09	0.98	1.7
	DAM	99.0	99.03	1.0	7.64	7.18	92.36	0.97	1.7
	SMALI	98.76	98.82	1.24	11.94	7.88	88.06	1.18	2.09
**EfficientNetB5**	APK	97.75	97.77	2.25	16.8	17.32	83.2	2.23	3.85
	AM	98.79	98.84	1.21	10.1	7.7	89.9	1.16	2.02
	CD	98.77	98.78	1.23	8.53	8.89	91.47	1.22	2.09
	DAM	98.86	98.94	1.14	9.18	5.47	90.82	1.06	1.89
	SMALI	98.48	98.56	1.52	13.41	9.45	86.59	1.44	2.55
**EfficientNetB6**	APK	97.73	97.78	2.27	19.67	16.77	80.33	2.22	3.85
	AM	98.04	98.09	1.96	18.02	14.55	81.98	1.91	3.33
	CD	98.86	98.85	1.14	7.72	8.36	92.28	1.15	1.96
	DAM	98.67	98.77	1.33	11.2	7.4	88.8	1.23	2.22
	SMALI	97.9	97.95	2.1	19.0	16.34	81.0	2.05	3.59
**EfficientNetB7**	APK	97.56	97.59	2.44	19.99	19.13	80.01	2.41	4.18
	AM	98.46	98.56	1.54	13.23	9.47	86.77	1.44	2.55
	CD	98.64	98.68	1.36	10.46	8.84	89.54	1.32	2.28
	DAM	98.59	98.68	1.41	11.5	7.85	88.5	1.32	2.35
	SMALI	98.41	98.46	1.59	13.02	10.26	86.98	1.54	2.68
**InceptionResNetV2**	APK	90.16	91.75	9.84	75.97	7.7	24.03	8.25	15.4
	AM	92.58	92.88	7.42	63.19	6.2	36.81	7.12	12.4
	CD	95.02	95.43	4.98	39.76	3.98	60.24	4.57	7.96
	DAM	92.69	93.11	7.31	55.09	6.2	44.91	6.89	12.4
	SMALI	93.23	93.87	6.77	51.35	5.61	48.65	6.13	11.23
**InceptionV3**	APK	95.54	95.63	4.46	36.22	31.92	63.78	4.37	7.51
	AM	98.05	98.15	1.95	17.63	11.91	82.37	1.85	3.26
	CD	98.78	98.79	1.22	9.19	9.18	90.81	1.21	2.09
	DAM	98.52	98.58	1.48	11.45	9.53	88.55	1.42	2.48
	SMALI	97.8	97.88	2.2	19.64	15.99	80.36	2.12	3.72
**MobileNet**	APK	96.34	96.4	3.66	33.73	24.26	66.27	3.6	6.2
	AM	99.1	99.14	0.9	7.26	5.37	92.74	0.86	1.5
	CD	98.74	98.74	1.26	9.51	9.83	90.49	1.26	2.15
	DAM	98.52	98.59	1.48	12.53	8.17	87.47	1.41	2.48
	SMALI	96.97	97.13	3.03	27.89	20.01	72.11	2.87	5.03
**MobileNetV2**	APK	97.03	97.11	2.97	25.48	17.84	74.52	2.89	4.96
	AM	98.74	98.78	1.26	11.49	10.19	88.51	1.22	2.15
	CD	98.5	98.55	1.5	13.51	9.43	86.49	1.45	2.55
	DAM	98.52	98.57	1.48	11.89	8.75	88.11	1.43	2.48
	SMALI	97.33	97.49	2.67	25.41	18.07	74.59	2.51	4.44

**Model**	**Format**	**TNR**	**NPV**	**FPR**	**FNR**	**FDR**	**TPR**	**FOR**	**MR**
**MobileNetV3Large**	APK	97.47	97.55	2.53	19.6	17.52	80.4	2.45	4.24
	AM	99.15	99.18	0.85	7.41	5.47	92.59	0.82	1.44
	CD	98.99	99.0	1.01	6.59	6.8	93.41	1.0	1.7
	DAM	98.99	99.03	1.01	8.43	6.97	91.57	0.97	1.7
	SMALI	98.5	98.55	1.5	13.3	12.03	86.7	1.45	2.55
**MobileNetV3Small**	APK	97.33	97.42	2.67	24.15	19.29	75.85	2.58	4.5
	AM	98.98	99.05	1.02	9.75	5.57	90.25	0.95	1.7
	CD	99.01	99.02	0.99	7.21	6.99	92.79	0.98	1.7
	DAM	98.95	98.99	1.05	8.68	6.34	91.32	1.01	1.76
	SMALI	98.53	98.58	1.47	12.4	9.83	87.6	1.42	2.48
**Xception**	APK	95.63	95.75	4.37	37.78	41.11	62.22	4.25	7.44
	AM	98.43	98.48	1.57	14.21	12.04	85.79	1.52	2.68
	CD	98.72	98.76	1.28	10.91	9.34	89.09	1.24	2.15
	DAM	98.68	98.68	1.32	10.25	11.07	89.75	1.32	2.28
	SMALI	97.33	97.47	2.67	25.52	18.95	74.48	2.53	4.44
