# Supplementary material for: Android malware analysis in a nutshell
Source: PLoS One. 2022 Jul 5;17(7):e0270647. doi: 10.1371/journal.pone.0270647 (PMC9255778; doi:10.1371/journal.pone.0270647)
Supplement: S2 Table — (PDF) [file pone.0270647.s002.pdf]

Table 11. Security performance of models on AMD dataset based on other metrics

Model	Format	TNR	NPV	FPR	FNR	FDR	TPR	FOR	MR
**Scratch**	APK	98.27	98.39	1.73	12.36	9.74	87.64	1.61	2.74
	AM	99.04	99.13	0.96	7.13	4.63	92.87	0.87	1.52
	CD	99.29	99.27	0.71	4.87	4.97	95.13	0.73	1.21
	DAM	99.61	99.63	0.39	2.67	2.3	97.33	0.37	0.63
	SMALI	98.85	98.8	1.15	6.82	7.38	93.18	1.2	1.98
**VGG16**	APK	97.5	97.6	2.5	17.69	14.71	82.31	2.4	4.04
	AM	98.82	98.83	1.18	7.83	7.41	92.17	1.17	1.94
	CD	98.98	99.03	1.02	7.08	5.73	92.92	0.97	1.64
	DAM	98.58	98.66	1.42	9.03	7.4	90.97	1.34	2.25
	SMALI	98.57	98.61	1.43	9.78	8.82	90.22	1.39	2.34
**ResNet50**	APK	97.83	97.91	2.17	14.96	13.02	85.04	2.09	3.51
	AM	98.86	98.92	1.14	7.52	6.44	92.48	1.08	1.82
	CD	99.2	99.2	0.8	5.42	5.12	94.58	0.8	1.33
	DAM	99.01	99.1	0.99	7.07	4.98	92.93	0.9	1.56
	SMALI	98.7	98.73	1.3	8.68	7.9	91.32	1.27	2.12
**VGG19**	APK	97.77	97.89	2.23	15.75	12.78	84.25	2.11	3.55
	AM	98.88	98.93	1.12	7.52	6.61	92.48	1.07	1.8
	CD	99.06	99.06	0.94	6.13	5.8	93.87	0.94	1.56
	DAM	98.48	98.52	1.52	9.38	9.0	90.62	1.48	2.45
	SMALI	98.44	98.54	1.56	11.07	9.27	88.93	1.46	2.5
**DenseNet121**	APK	96.96	97.12	3.04	20.73	16.87	79.27	2.88	4.82
	AM	98.55	98.65	1.45	10.31	8.16	89.69	1.35	2.3
	CD	98.99	99.03	1.01	6.77	5.91	93.23	0.97	1.64
	DAM	98.72	98.75	1.28	8.73	8.07	91.27	1.25	2.1
	SMALI	98.34	98.35	1.66	10.56	9.96	89.44	1.65	2.72
**DenseNet169**	APK	97.01	97.2	2.99	21.06	16.94	78.94	2.8	4.74
	AM	98.48	98.62	1.52	10.98	8.69	89.02	1.38	2.41
	CD	98.98	99.03	1.02	7.06	5.87	92.94	0.97	1.64
	DAM	98.52	98.6	1.48	10.11	8.7	89.89	1.4	2.37
	SMALI	98.38	98.41	1.62	11.07	9.71	88.93	1.59	2.64
**DenseNet201**	APK	97.66	97.77	2.34	16.36	13.6	83.64	2.23	3.75
	AM	98.12	98.37	1.88	13.7	9.17	86.3	1.63	2.89
	CD	98.93	98.98	1.07	7.23	5.99	92.77	1.02	1.72
	DAM	98.58	98.61	1.42	8.97	8.7	91.03	1.39	2.31
	SMALI	98.49	98.51	1.51	10.07	8.98	89.93	1.49	2.46
**EfficientNetB0**	APK	97.8	97.93	2.2	15.99	12.51	84.01	2.07	3.51
	AM	98.54	98.65	1.46	10.6	8.83	89.4	1.35	2.32
	CD	99.02	99.06	0.98	6.94	6.13	93.06	0.94	1.6
	DAM	98.0	98.31	2.0	14.7	8.7	85.3	1.69	3.03
	SMALI	98.26	98.37	1.74	12.7	10.06	87.3	1.63	2.78
**EfficientNetB1**	APK	98.01	98.1	1.99	14.38	11.66	85.62	1.9	3.21
	AM	98.65	98.72	1.35	9.45	8.0	90.55	1.28	2.16
	CD	99.02	99.02	0.98	6.15	6.04	93.85	0.98	1.62
	DAM	98.27	98.41	1.73	11.78	8.97	88.22	1.59	2.71
	SMALI	98.4	98.38	1.6	10.98	10.33	89.02	1.62	2.7
**EfficientNetB2**	APK	97.59	97.74	2.41	18.39	14.46	81.61	2.26	3.87
	AM	98.44	98.51	1.56	10.71	9.24	89.29	1.49	2.51
	CD	98.87	98.9	1.13	7.74	6.73	92.26	1.1	1.84
	DAM	98.6	98.69	1.4	9.54	7.93	90.46	1.31	2.22
	SMALI	98.26	98.37	1.74	12.87	9.98	87.13	1.63	2.8

Model	Format	TNR	NPV	FPR	FNR	FDR	TPR	FOR	MR
**EfficientNetB3**	APK	97.68	97.91	2.32	17.92	12.34	82.08	2.09	3.65
	AM	98.63	98.72	1.37	9.91	7.59	90.09	1.28	2.18
	CD	99.02	99.0	0.98	6.05	6.35	93.95	1.0	1.64
	DAM	98.71	98.79	1.29	8.55	6.82	91.45	1.21	2.04
	SMALI	98.32	98.34	1.68	11.74	11.22	88.26	1.66	2.78
**EfficientNetB4**	APK	98.1	98.22	1.9	14.04	11.13	85.96	1.78	3.05
	AM	98.26	98.46	1.74	12.93	8.91	87.07	1.54	2.71
	CD	98.99	99.01	1.01	6.93	6.2	93.07	0.99	1.66
	DAM	98.57	98.68	1.43	9.41	7.86	90.59	1.32	2.25
	SMALI	98.51	98.49	1.49	10.41	10.28	89.59	1.51	2.52
**EfficientNetB5**	APK	97.7	97.91	2.3	16.91	12.21	83.09	2.09	3.61
	AM	98.31	98.43	1.69	11.88	9.84	88.12	1.57	2.69
	CD	99.06	99.08	0.94	6.39	5.9	93.61	0.92	1.54
	DAM	98.48	98.51	1.52	9.7	9.15	90.3	1.49	2.47
	SMALI	98.33	98.38	1.67	11.71	10.36	88.29	1.62	2.72
**EfficientNetB6**	APK	97.97	98.17	2.03	15.11	10.59	84.89	1.83	3.19
	AM	98.16	98.25	1.84	13.07	11.69	86.93	1.75	2.97
	CD	98.91	98.94	1.09	7.32	6.61	92.68	1.06	1.78
	DAM	98.67	98.65	1.33	7.93	8.35	92.07	1.35	2.2
	SMALI	98.04	98.03	1.96	13.22	12.47	86.78	1.97	3.31
**EfficientNetB7**	APK	97.89	98.05	2.11	15.73	11.94	84.27	1.95	3.35
	AM	98.69	98.77	1.31	9.43	8.03	90.57	1.23	2.1
	CD	99.04	99.07	0.96	6.95	5.96	93.05	0.93	1.58
	DAM	98.69	98.79	1.31	8.86	6.59	91.14	1.21	2.06
	SMALI	98.3	98.3	1.7	11.77	11.38	88.23	1.7	2.85
**InceptionResNetV2**	APK	91.94	92.81	8.06	63.5	6.53	36.5	7.19	13.05
	AM	94.11	94.99	5.89	47.59	4.44	52.41	5.01	8.87
	CD	95.87	95.84	4.13	27.16	3.51	72.84	4.16	7.01
	DAM	91.61	92.01	8.39	55.75	6.61	44.25	7.99	13.23
	SMALI	92.7	93.52	7.3	60.31	5.65	39.69	6.48	11.3
**InceptionV3**	APK	95.16	95.23	4.84	25.65	26.51	74.35	4.77	8.54
	AM	97.91	98.0	2.09	14.67	12.87	85.33	2.0	3.38
	CD	98.86	98.91	1.14	7.97	6.48	92.03	1.09	1.84
	DAM	98.29	98.38	1.71	11.14	10.51	88.86	1.62	2.73
	SMALI	97.13	97.31	2.87	20.42	18.04	79.58	2.69	4.58
**MobileNet**	APK	96.8	96.99	3.2	22.46	19.84	77.54	3.01	5.1
	AM	98.51	98.52	1.49	9.58	9.09	90.42	1.48	2.45
	CD	98.68	98.69	1.32	8.64	7.93	91.36	1.31	2.16
	DAM	98.22	98.4	1.78	12.14	9.13	87.86	1.6	2.77
	SMALI	97.26	97.39	2.74	19.1	16.67	80.9	2.61	4.4
**MobileNetV2**	APK	96.81	96.97	3.19	22.29	18.66	77.71	3.03	5.1
	AM	98.26	98.41	1.74	12.87	9.56	87.13	1.59	2.75
	CD	98.52	98.54	1.48	10.13	9.05	89.87	1.46	2.45
	DAM	98.32	98.38	1.68	11.31	10.01	88.69	1.62	2.71
	SMALI	97.29	97.29	2.71	17.6	15.48	82.4	2.71	4.5

Model	Format	TNR	NPV	FPR	FNR	FDR	TPR	FOR	MR
**MobileNetV3Large**	APK	97.72	97.83	2.28	15.64	13.35	84.36	2.17	3.65
	AM	98.63	98.65	1.37	9.06	8.47	90.94	1.35	2.24
	CD	99.03	99.01	0.97	6.0	6.29	94.0	0.99	1.62
	DAM	98.82	98.94	1.18	8.1	5.58	91.9	1.06	1.82
	SMALI	98.55	98.57	1.45	10.49	9.19	89.51	1.43	2.4
**MobileNetV3Small**	APK	97.69	97.81	2.31	16.48	13.23	83.52	2.19	3.71
	AM	98.64	98.65	1.36	8.72	8.51	91.28	1.35	2.22
	CD	99.06	99.04	0.94	5.94	6.28	94.06	0.96	1.58
	DAM	98.73	98.73	1.27	7.85	7.87	92.15	1.27	2.08
	SMALI	98.47	98.49	1.53	10.33	9.56	89.67	1.51	2.52
**Xception**	APK	96.29	96.44	3.71	25.11	22.02	74.89	3.56	5.93
	AM	98.36	98.46	1.64	11.57	9.06	88.43	1.54	2.61
	CD	98.53	98.51	1.47	9.27	9.16	90.73	1.49	2.45
	DAM	98.5	98.54	1.5	9.65	9.19	90.35	1.46	2.43
	SMALI	97.42	97.49	2.58	17.79	15.82	82.21	2.51	4.18
